# AtGSTU19 and AtGSTU24 as Moderators of the Response of *Arabidopsis thaliana* to *Turnip mosaic virus*

**DOI:** 10.3390/ijms231911531

**Published:** 2022-09-29

**Authors:** Katarzyna Otulak-Kozieł, Edmund Kozieł, Edit Horváth, Jolán Csiszár

**Affiliations:** 1 Institute of Biology, Department of Botany, Warsaw University of Life Sciences, Nowoursynowska 159, 02-776 Warsaw, Poland; 2 Department of Plant Biology, Faculty of Science and Informatics, University of Szeged, Közěp fasor 52, H-6726 Szeged, Hungary

**Keywords:** glutathione, glutathione S-transferase *tau* class, plant–virus interaction, plant cell ultrastructure

## Abstract

Plants produce glutathione as a response to the intercellular redox state. Glutathione actively participates in the reactive oxygen species (ROS)-dependent signaling pathway, especially under biotic stress conditions. Most of the glutathione S-transferases (GSTs) are induced in cells during the defense response of plants not only through highly specific glutathione-binding abilities but also by participating in the signaling function. The tau class of GSTs has been reported to be induced as a response under stress conditions. Although several studies have focused on the role of the tau class of GSTs in plant–pathogen interactions, knowledge about their contribution to the response to virus inoculation is still inadequate. Therefore, in this study, the response of *Atgstu19* and *Atgstu24* knockout mutants to mechanical inoculation of *Turnip mosaic virus* (TuMV) was examined. The systemic infection of TuMV was more dynamically promoted in Atgstu19 mutants than in wild-type (Col-0) plants, suggesting the role of GSTU19 in TuMV resistance. However, *Atgstu24* mutants displayed virus limitation and downregulation of the relative expression of TuMV capsid protein, accompanied rarely by TuMV particles only in vacuoles, and ultrastructural analyses of inoculated leaves revealed the lack of virus cytoplasmic inclusions. These findings indicated that Atgstu24 mutants displayed a resistance-like reaction to TuMV, suggesting that GSTU24 may suppress the plant resistance. In addition, these findings confirmed that GSTU1 and GSTU24 are induced and contribute to the susceptible reaction to TuMV in the *Atgstu19*–TuMV interaction. However, the upregulation of GSTU19 and GSTU13 highly correlated with virus limitation in the resistance-like reaction in the *Atgstu24*–TuMV interaction. Furthermore, the highly dynamic upregulation of GST and glutathione reductase (GR) activities resulted in significant induction (between 1 and 14 days post inoculation [dpi]) of the total glutathione pool (GSH + GSSG) in response to TuMV, which was accompanied by the distribution of active glutathione in plant cells. On the contrary, in *Atgstu19*, which is susceptible to TuMV interaction, upregulation of GST and GR activity only up to 7 dpi symptom development was reported, which resulted in the induction of the total glutathione pool between 1 and 3 dpi. These observations indicated that GSTU19 and GSTU24 are important factors in modulating the response to TuMV in *Arabidopsis thaliana*. Moreover, it was clear that glutathione is an important component of the regulatory network in resistance and susceptible response of *A. thaliana* to TuMV. These results help achieve a better understanding of the mechanisms regulating the Arabidopsis–TuMV pathosystem.

## 1. Introduction

Glutathione (in reduced form—GSH) is a ubiquitous thiol in eukaryotes. GSH plays a vital role in plant development [1] and mediates important cellular processes, such as programmed cell death [2]. Moreover, it is one of the most important cellular antioxidants, as it scavenges reactive oxygen species (ROS), which are by-products of aerobic metabolism [3]. Therefore, glutathione is considered a crucial metabolite in plants’ response to both abiotic and biotic stress, where it scavenges ROS and limits the extent of oxidative damage [4]. Furthermore, it is not only a simple antioxidant but also involved in redox-sensitive protein control, as it can change the intercellular redox state to the defense response of plants through the ROS-dependent signaling pathway [5,6]. In the literature, the role of glutathione in plant defense and tolerance against microorganisms, such as bacteria [7,8,9] and fungi [10,11,12], has been described in detail. Several authors have reported the participation of glutathione in plants’ physiological and biochemical response to virus infection [13,14,15,16]. In addition, high GSH accumulation reduced the coat protein content of Tobacco mosaic virus (TMV) and necrotic symptoms, as reported by Gullner et al. [17]. In general, increases in GSH and GSH-related enzymes were significantly correlated with resistance and signaling under biotic stress conditions [18]. Moreover, our previous studies confirmed that glutathione was significantly differentially distributed in plant cells during potato virus Y (PVY^NTN^)–potato interaction [16]. Furthermore, a steady induction of two glutathione S-transferases (GSTs)—StGSTF2 and StGSTF1—from the *phi* class in Solanum tuberosum, regardless of interaction type was reported, among which StGSTF2 acts as a marker of resistance response in hypersensitive reaction to PVY^NTN^.

GSTs (E.C. 2.5.1.18) play the role of catalysts in GSH-dependent reactions, such as conjugation to substrates, hydroxyperoxidase activity, and dehydroascorbate reductase activity [19,20]. Several reports have indicated that the expression and activity of GSTs are dependent on the GSH content and the GSH/GSSG ratio. The transcription of GST genes may be upregulated by highly oxidizing environments, high GSSG levels, and low GSH levels [21]. GSTs also play a noncatalytic role in the transport and distribution of a wide range of ligands to specific receptors or compartments [22]. Recent plant genome analyses have shown that plant GSTs can be divided into 14 classes, with the *tau* (GSTU) and *phi* (GSTF) classes being the largest groups [23]. Among the 52 GSTs in Arabidopsis, 42 are classified as GSTUs and GSTFs [22,24]. Studies have confirmed that most of the GSTs are induced by abiotic and biotic stress and that they play positive roles in defense response [25]. GSTUs and GSTFs, due to their high specificity to the GSH-binding region, are also involved in the signaling function [26]. The expression of GSTUs and GSTFs is often reported as a response to environmental stresses, such as metal treatment [27], salinity, cold, drought, and heat [28,29,30]. The induction of GSTUs in biotic interactions, such as with pathogenic responses, has also been reported [19,31]. Although 28 GSTs of *Arabidopsis thaliana* belong to the GSTU class, gaps in knowledge about the functions of GSTUs in plant stress response still remain. Moreover, recent studies focus mainly on the role of GSTUs under abiotic stress conditions [32]. GSTU13 plays a catalytic role in the conjugation of GSH, affecting the immune response of *A. thaliana* to fungal pathogens *Eryshiphe pisi*, *Colletotrichum*, and *Plectosphaerella* [33]. Moreover, gstu13 mutants display enhanced susceptibilities in this pathosystem. In addition, Pantelides et al. [34] have reported that AtGSTU16, another *tau* class member, is a part of the response to *Verticillium dahliae*. Although some information on the involvement of individual AtGSTUs in plant response to the pathogen is available, their role in plant–virus interactions is not adequately investigated. We chose *Turnip mosaic virus* (TuMV), which is the member of the largest family of RNA plant viruses—*Potyviridae* as a model in studies plant–virus interaction [35]. In this study, the putative contribution of AtGSTU19 and AtGSTU24 in response to TuMV (Potyvirus) inoculation was analyzed. Under control conditions, AtGSTU19 represents a significant percentage of the GST pool [36]. Moreover, *Atgstu19* knockdown mutants have only been analyzed under salt treatments so far [37]. Furthermore, Atgstu19 was found to be expressed in almost all investigated plant tissues and had the highest relative transcription level among AtGSTs [38]. In addition, AtGSTU19 may act as a stress regulator by increasing the activities of antioxidant enzymes, leading to ROS scavenging activity and maintaining homeostasis [39]. However, only one study on the role of GSTU19 in plant pathogen response has been found in the literature: Wagner et al. [40], who reported that GSTU19 was induced in interactions with *Peronospora parasitica*. Taking into account data presented by Wagner et al. [40] of inoculating Arabidopsis plants with the downy mildew pathogen *Peronospora parasitica* isolate NOCO (compatible interaction) and *P. parasitica* isolate EMWA (incompatible interaction), expression of the AtGSTs was compared to the expression of the pathogenesis related protein (PR1), which can act as a marker for defense gene induction. In the compatible interaction, the expression of *AtGSTU19* (with GSTF2 that was previously analyzed by us) was found to be co-induced with PR1 three days after inoculation. Whereas in the incompatible interaction, the AtGSTFs showed much weaker or even no induction at all, while *AtGSTU19* was transiently induced two days after infection, which preceded the induction of PR1 at 3 dpi. Therefore, we can surmise that *AtGSTU19* can contribute in virus biotic stress as well, as we were intrigued how these plants would react to potyvirus inoculation.

Moreover, knowledge about AtGSTU24 under stress conditions is much poorer. GSTU24 transcripts are strongly induced by treatments with a wide range of xenobiotics [41,42] and lipid stress response [43]. Knockout *Atgstu24* mutant lines are redox homeostasis regulators as an effect of salt stress [37].

In the present study, for the first time, the response of *Atgstu19* and *Atgstu24* knockout mutants to TuMV pathogenic stress was examined. Significant differences were observed in the expression of the selected *Atgstu* genes, which indicated the specific contribution of individual AtGSTUs in different reactions to TuMV. Moreover, as the limitation virus content was highly dynamic, the induction of the total glutathione pool, accompanied by the upregulation of the activity GSTs and glutathione reductases (GRs), correlated with resistance-like reaction in the *Atgstu24*–TuMV interaction. On the contrary, significant changes in the distribution of glutathione in plant cells and glutathione pool induction of up to just 3 days post-inoculation (dpi) after TuMV infection showed susceptible *Atgstu19*–TuMV interaction. Moreover, these findings revealed the importance of selected AtGSTUs in the regulation of TuMV infection in Arabidopsis.

## 2. Results

### 2.1. Different TuMV Concentrations Correlated with the Ultrastructural Response of Plants

**Figure 1:**
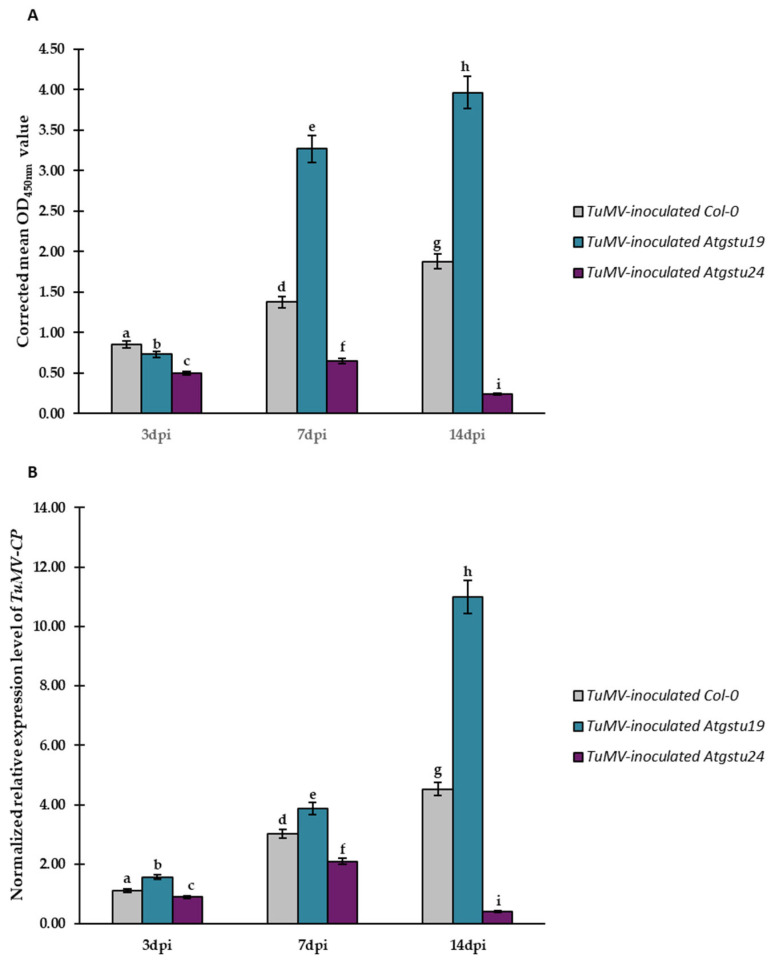
Validation of TuMV concentration in Col-0, *Atgstu19*, and *Atgstu24* plants at 3, 7, and 14 dpi by quantitative DAS-ELISA (**A**) and normalized relative expression of *TuMV-CP* (**B**). (**A**) DAS-ELISA detection of TuMV. Values represent corrected mean OD_405 nm_. (**B**) Normalized relative expression of *TuMV-CP* calculated based on the mean expression of *AtEf1α* and *AtF-box* reference genes. Statistical significance of differences was assessed at the *p* < 0.05 level using ANOVA with post hoc Tukey’s HSD (indicated by letters above the bars).

To examine plants’ reaction to TuMV inoculation, Col-0 and two GST tau class gene mutants (*Atgstu19* and *Atgstu24*) were selected. Wild-type Col-0 displayed susceptibility to TuMV isolate PV-0104 [44]. The double-antibody sandwich enzyme-linked immunosorbent assay (DAS-ELISA) performed on the samples collected between 3 and 14 dpi confirmed the presence of TuMV in all virus-inoculated plants. Moreover, TuMV was not detected in mock-inoculated Col-0 and mutant plants (Table S1). OD_405 nm_ values were significantly higher in *Atgstu19* plants than in *Atgstu24* plants and susceptible Col-0 plants by 3 to 14 dpi (Table S1). The corrected mean OD_405 nm_ values showed a statistically significant increase in the relative concentrations of TuMV in Col-0 (1.61-fold between 3 and 7 dpi and 1.37-fold between 7 and 14 dpi) and also in *Atgstu19* (4.47-fold between 3 and 7 dpi and 1.21-fold between 7 and 14 dpi) plants. However, in TuMV-inoculated *Atgstu24* plants, the corrected mean OD_405 nm_ values only indicated a significant increase in virus concentrations between 3 and 7 dpi (approximately 1.31-fold) and a high level of decrease in virus concentrations between 7 and 14 dpi (2.74-fold; Figure 1A). This observation was confirmed by the validation of the normalized expression of the TuMV capsid protein (*TuMV-CP*) gene, which was analyzed based on two plant host reference genes—*AtEf1α* and *AtF-Box*—to show changes in TuMV concentrations in the inoculated leaves (Figure 1B). DAS-ELISA results showed that the expression of *TuMV-CP* changed similarly to the relative concentration of TuMV. Moreover, the expression of *TuMV-CP* was upregulated in Col-0 (2.68-fold between 3 and 7 dpi and 1.50-fold between 7 and 14 dpi) and in *Atgstu19* (2.48-fold between 3 and 7 dpi and 2.84-fold between 7 and 14 dpi) plants. Furthermore, the TuMV concentration and the dynamics of the increase in TuMV-CP expression were much higher in *Atgstu19* plants than in Col-0 plants. However, in virus-inoculated *Atgstu24* plants, *TuMV-CP* expression indicated an increase in virus concentration between 3 and 7 dpi (2.32-fold) and a high reduction in virus concentration between 7 and 14 dpi (5.24-fold; Figure 1B), even lower than the levels measured at the 3 dpi time point.

Taken together, the results of the relative virus concentration and the normalized expression of *TuMV-CP* showed that *Atgstu19* plants displayed more susceptible reactions than Col-0 plants. This also suggested that *Atgstu24* plants presented a different, resistant-like reaction to TuMV, in comparison with *Atgstu19* and Col-0 plants.

**Figure 2:**
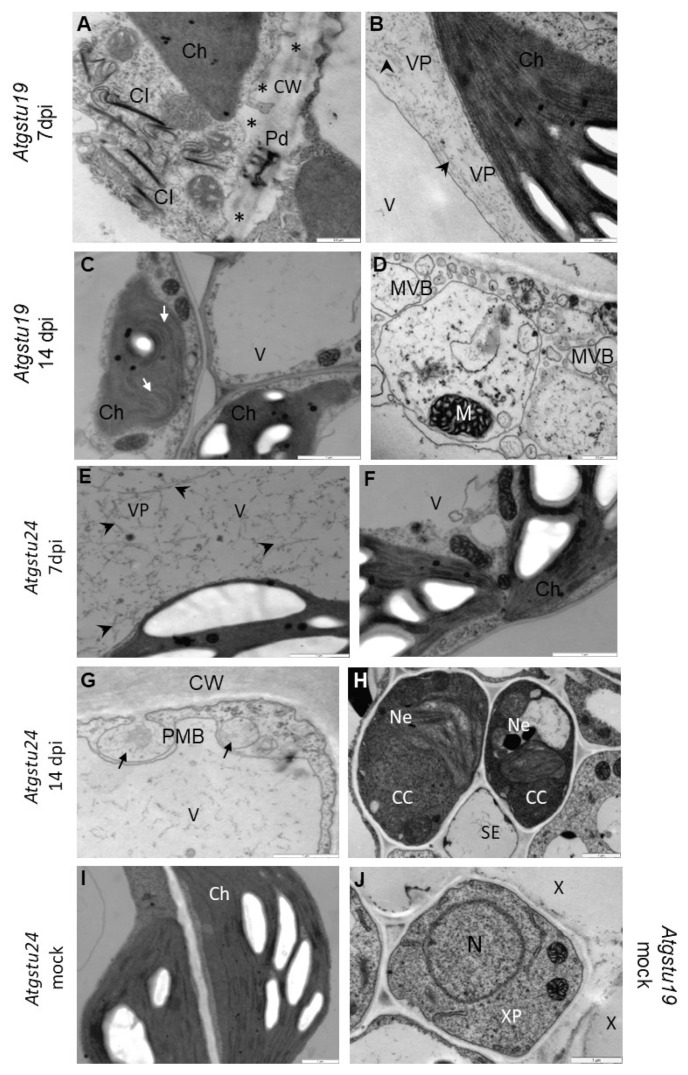
Ultrastructural alterations in *Atgstu19* (**A**–**D**) and *Atgstu24* (**E**–**H**) in response to TuMV at 7 (**A**,**B**,**E**,**F**) and 14 (**C**,**D**,**G**,**H**) days after inoculation and mock inoculated plants (**I**,**J**). (**A**) TuMV cytoplasmic inclusion (CI) in mesophyll cell. Changed (*) cell wall (CW) structure in plasmodesmata (Pd) area. Scale bar 0.5 μm. (**B**) Virus particles (VP, arrowhead) in cytoplasm of mesophyll cell. Scale bar 0.5 μm. (**C**) Curved thylacoids (arrow) in chloroplasts (Ch) of spongy mesophyll cells. Scale bar 1 μm. (**D**) Multivesicular bodies (MVB) in phloem parenchyma cell. Scale bar 0.5 μm. (**E**) Virus particles (VP, arrowheads) in vacuole of mesohyll cell. Scale bar 1 μm. (**F**) Unchanged chloroplast thyalcoids. Scale bar 1 μm. (**G**) Paramular bodies (arrows, PMB) in cytoplasm of epidermis. Scale bar 1 μm. (**H**) Necroses (Ne) in phloem companion cells (CC). Scal bar 1 μm. (**I**) Ultrastructure of mock-inoculated mesohyll cells. Scale bar 1 μm. (**J**) Ultrastructure of mock-inoculated xylem elements. Scale bar 1 μm. CC—companion cell, Ch—chloroplast, M—mitochondria, N—nucleus, Ne—necrosis, SE—sieve element, V—vacuole, VP—virus particles, X—xylem tracheary elements, XP—xylem parenchyma.

The reaction of virus-inoculated Col-0 (Figure S1B(B’,C’)), *Atgstu19* mutant, *Atgstu24* mutant (Figure 2), and mock-inoculated plants (Figure S1B(A’) and Figure 2I,J) was examined using ultrastructural analysis. Interestingly, virus cytoplasmic inclusions accompanied by TuMV were observed primarily in *Atgstu19* tissues (Figure 2A–D) and in Col-0 starting from 7 dpi (Figure S1B(B’,C’)). Moreover, curved chloroplast thylakoids, changes in the cell wall structure, and multivesicular bodies were highly induced in the TuMV–*Atgstu19* interaction (Figure 2A,C,D). *Atgstu19* mutants showed significantly higher virus alterations than Col-0 plants. On the contrary, in *Atgstu24* mutants, virus particles were observed in lower frequency and only in vacuoles (Figure 2E). Neither virus cytoplasmic inclusions nor changes in chloroplast thylakoids were observed in the TuMV–*Atgstu24* interaction (Figure 2F). However, phloem necrotic changes and the induction of paramular bodies (PMB), especially in the epidermis, were observed (Figure 2G,H). These observations indicate that *Atgstu24* plants displayed far fewer virus particles than *Atgstu19* and Col-0 plants.

**Figure 3:**
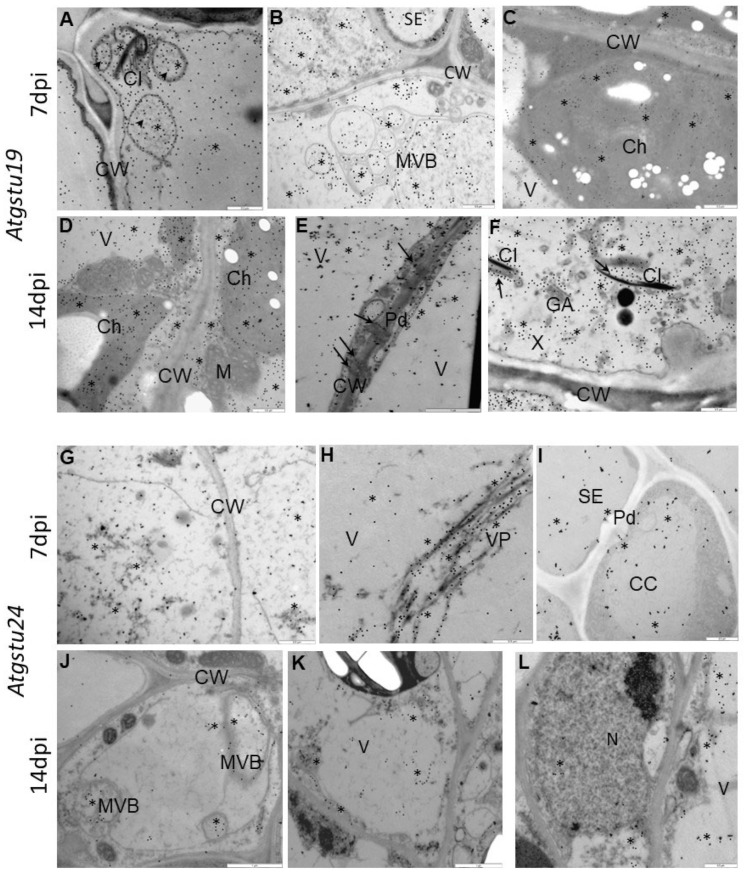
Immunogold labeling of TuMV in *Atgstu19* (**A**–**F**) and *Atgstu24* (**G**–**L**) 7 (**A**–**C**,**G**–**I**) and 14 (**C**–**E**,**J**–**L**) days after inoculation. (**A**) TuMV epitope deposition (*) round cytoplasmic virus inclusions (CI) and vesicular structures (arrowheads) in pallisade mesophyll cell. Scale bar 0.5 μm. (**B**) TuMV epitope deposition (*) in multivesicular bodies (MVB) of phloem parenchyma cells. TuMV eptope deposits in cytoplasm and vacuole (V). Scale bar 0.5 μm. (**C**) Gold particles (*) in chloroplasts (Ch) and vacuole (V). Scale bar 0.5 μm. (**D**) TuMV epitope deposition (*) indicating TuMV presence in chloroplasts (Ch), along the cell wall (CW) and in vacuole (V). Scale bar 0.5 μm. (**E**) TuMV epitope deposition (*) along cell wall (CW) and inside plasmodesmata (Pd, arrows) between phloem cells. Scale bar 1 μm. (**F**) TuMV epitope deposition (*) inside xylem tracheaery elements. Virus inclusions (CI, arrows) inside xylem tracheary element (X). Scale bar 0.5 μm. (**G**) TuMV epitope deposition (*) in cytoplasm of mesophyll cell. Scale bar 0.5 μm. (**H**) TuMV epitope deposition (*) along virus particles (VP) in vacuole (V) of mesohyll cell. Scale bar 0.5 μm. (**I**) TuMV epitope deposition (*) in sieve element (SE) and vacuole (V) of phloem cells. Scale bar 0.5 μm. (**J**) TuMV (*) around multivesicular bodies (MVB) in phloem cell. Scale bar 1 μm. (**K**) TuMV epitope deposition (*) in vacuole (V) of phloem parenchyma cells. Scale bar 1 μm. (**L**) TuMV epitope deposition (*) in vacuoles of spongy mesophyll cell. A few gold granules in nucleus (N). Scale bar 0.5 μm. CC—companion cell, GA—trans-Golgi network, Pd—plasmodesmata, SE—sieve elements, X—xylem tracheary elements.

**Figure 4:**
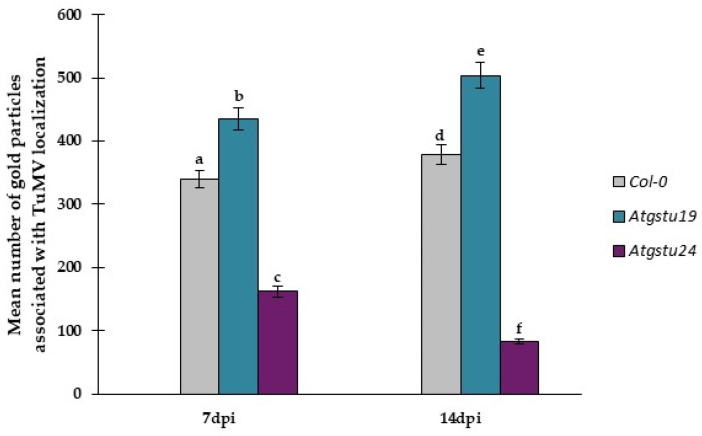
Quantification of gold particles associated with TuMV in *Arabidopsis thaliana* Col-0, *Atgstu19*, and *Atgstu24* mutants at 7 and 14 dpi. Significant differences between classes at the *p* < 0.05 level were assessed by ANOVA with post hoc Tukey’s HSD. Statistically significant values are indicated by letters above chart bars.

Besides virus concentration and plant tissue changes, in situ TuMV localization in Col-0, *Atgstu19* mutants, and *Atgstu24* mutants was monitored at the ultrastructural level (Figure S1B(D’), Figure 3A–L and Figure 4). The quantification of gold particles associated with the TuMV epitope showed significant changes in virus distribution during infection (Figure 4). In Col-0 and *Atgstu19* mutants, the localization of the TuMV epitope was increased 1.11-fold and 1.16-fold, respectively, between 7 and 14 dpi. However, the level of localization was much higher in *Atgstu19* mutants than in Col-0 plants (Figure 3A–F, Figure 4 and Figure S1B(D’)). Seven days post-TuMV inoculation, depositions were observed around virus cytoplasmic inclusions, in chloroplasts (Figure 3A,C) in mesophyll cells, and in membranous/vesicular structures in vascular tissues (Figure 3B). Fourteen days post-inoculation, when more intense deposition was noticed, TuMV epitopes were observed in chloroplasts, in cytoplasm, and inside the plasmodesmata of mesophyll cells (Figure 3D,E). Strong localization was also observed inside xylem tracheary elements around virus inclusions (Figure 3F). These observations confirmed the systemic infection of the virus in *Atgstu19* and Col-0 plants. However, gold depositions were observed neither in control mock-inoculated tissues nor in tissue sections, where primary antibodies were replaced with the pre-immune serum.

On the contrary, the localization of the TuMV epitope confirmed the findings of the relative virus concentration analyses and was significantly lower in *Atgstu24* mutants than in *Atgstu19* and Col-0 plants and decreased (1.95-fold) between 7 and 14 dpi (Figure 3G–L and Figure 4). Moreover, in *Atgstu24* mutants, the localization of the TuMV epitope was observed primarily in vacuoles in mesophyll and vascular tissues 7 dpi and also around virus particles (Figure 3G–I). Furthermore, the localization at the 14 dpi time point in *Atgstu24* mutants around membranous structures and in vacuoles in mesophyll and phloem was less intense than at the 7 dpi time point (Figure 3J–L).

The relative virus concentration determined that using ultrastructural plant response analyses, along with the *TuMV-CP* expression and DAS-ELISA results, confirmed the TuMV infection limitation in the *Atgstu24*–TuMV interaction, in contrast to highly induced systemic infection, especially during the *Atgstu19*–TuMV interaction and also in Col-0 plants. Moreover, taking into account the *TuMV-CP* expression, relative virus concentration, and ultrastructural analyses, it can be summarized that AtGSTU19 is required to limit virus concentration, whereas AtGSTU24 seems to play a role in susceptible interaction.

### 2.2. Significant Changes in Reduced and Oxidized Glutathione Forms as an Important Factor for Susceptibility and Resistance-like Reaction in Atgstu19 or Atgstu24

**Figure 5:**
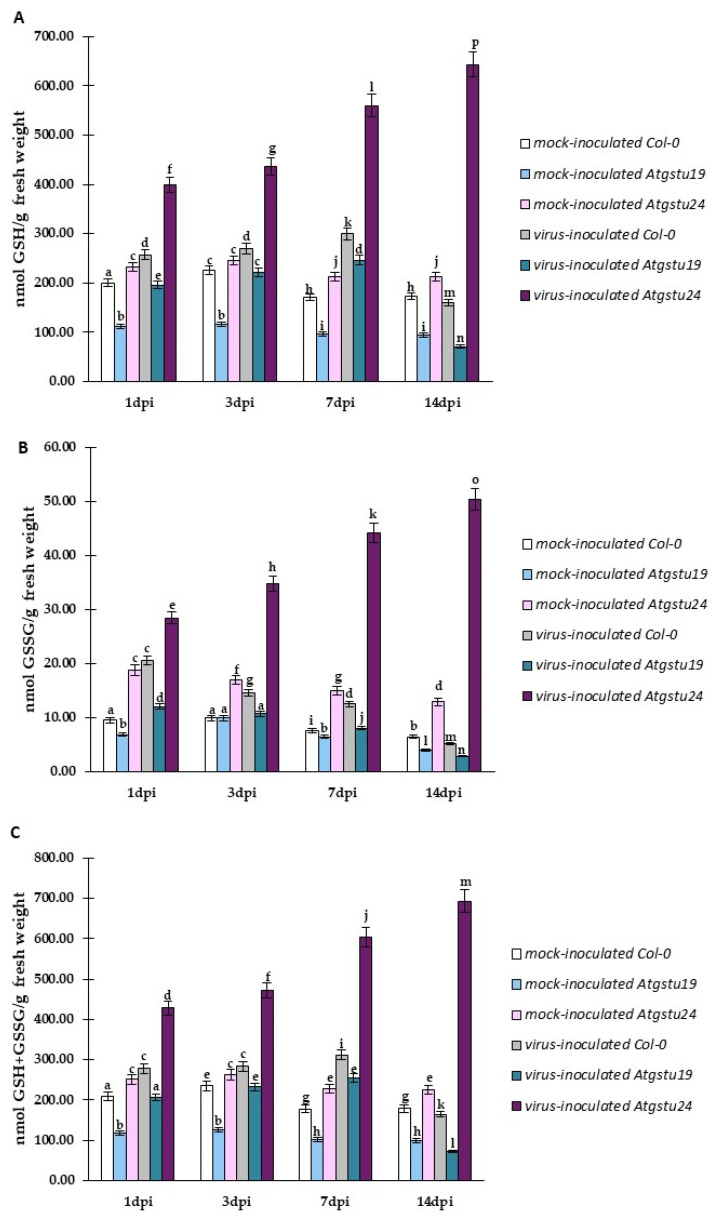
The mean concentration of GSH (**A**) and GSSG (**B**) glutathione in the leaves of TuMV and mock-inoculated Col-0, *Atgstu19*, and *Atgstu24* plants between 1 and 14 dpi. (**C**) The mean of the total concentration of GSH and GSSG glutathione in the leaves of TuMV- and mock-inoculated Col-0, *Atgstu19*, and *Atgstu24* plants between 1 and 14 dpi. Using ANOVA and Tukey’s HSD test, the mean concentrations of GSH and GSSG were calculated at *p* < 0.05. Statistically significant values are indicated by letters above the bars.

The changes in the *TuMV-CP* expression during viral infection in GSTU mutants highlighted the putative and crucial role of glutathione in the modulation of susceptibility to TuMV in *Atgstu19* and Col-0 plants, and resistance-like reaction to TuMV in *Atgstu24* plants. To evaluate this observation, high-performance liquid chromatography (HPLC) experiments were carried out to analyze the levels of different glutathione forms, such as reduced glutathione—GSH (Figure 5A), oxidized glutathione—GSSG (Figure 5B), and total glutathione content—GSH + GSSG (Figure 5C) after TuMV inoculation of Col-0, *Atgstu19*, and *Atgstu24* plants. HPLC analyses revealed distinctive modulation patterns of GSH and GSSG in Col-0, *Atgstu19*, and *Atgstu24* during viral infection. In general, TuMV infection increased the GSH content from 1 to 7 dpi, in comparison with mock-inoculated Col-0 and mutant plants. The highest GSH content was observed in virus-inoculated *Atgstu24* plants, which revealed a resistance-like reaction to TuMV (Figure 5A). Moreover, the GSH content in these plants was also continuously upregulated between 1 and 14 dpi (1.62-fold). This continuous upregulation of GSH after TuMV inoculation indicated that *Atgstu24* plants could more efficiently/precisely protect their cells from oxidative stress. However, in the virus-inoculated susceptible Col-0 and *Atgstu19* plants, the GSH content was increased only at the early time points between 1 and 7 dpi—up to TuMV symptoms appeared—and was reduced between 7 and 14 dpi (Figure 5A). Virus-inoculated Col-0 plants showed a 1.16-fold increase in the GSH content between 1 and 7 dpi and a 1.87-fold decrease at 7 dpi. Interestingly, the GSH content in TuMV–*Atgstu19* plants was even lower than in susceptible Col-0 plants. In TuMV–*Atgstu19* plants, the increase in the GSH content was 1.26-fold from 1 to 7 dpi, whereas the decrease at 14 dpi was more dynamic (about 3.07-fold) than in Col-0 plants. These results also revealed that 7 dpi is a crucial time point in the susceptible interaction between TuMV and Arabidopsis plants. All plants started showing problems in counteracting oxidative stress at 7 dpi. However, more severe problems in counteracting the oxidative stress were observed in the more susceptible *Atgstu19* plants.

The concentration of GSSG was modulated during TuMV infection (Figure 5B). In virus-inoculated *Atgstu24* plants, the GSSG concentration steadily increased 1.77-fold between 1 and 14 dpi and was much higher than in mock-inoculated plants. This also suggested that TuMV inoculation modulated the GSH concentration and resulted in the direct conversion of GSH to GSSG. This observation underlined that virus-inoculated *Atgstu24* plants showed precise protection against oxidative stress using upregulated levels of GSH, which was observed with the increased production of the GSSG form. However, in virus-inoculated susceptible Col-0 and *Atgstu19* plants, the GSSG concentration was downregulated 4-fold and 4.29-fold, respectively. This demonstrated the problems in counteracting the oxidative stress in Col-0 and *Atgstu19* plants. Further evaluation using HPLC analyses revealed that not only GSH and GSSG forms were modulated, but there were also changes in the total glutathione (GSH+GSSG) level (Figure 5C). In resistance-like reaction, TuMV–*Atgstu24* plants showed a 1.62-fold upregulation of the GSH+GSSG content between 1 and 14 dpi. In contrast, in TuMV–Col-0 (1.23-fold) and TuMV–*Atgstu19*-susceptible plants, a slight upregulation was observed only between 1 and 7 dpi, with a strong reduction between 7 and 14 dpi—1.89-fold for Col-0 and 3.48-fold for *Atgstu19*.

### 2.3. Subcellular Effect of Glutathione (GSH + GSSG) Distribution in Interactions between TuMV and Atgstu19, Atgstu24, and Col-0 Plants

Significant changes in GSH and GSSG concentrations indicated that the glutathione content was highly increased, especially in the TuMV–*Atgstu24* interaction, whereas in the TuMV–*Atgstu19* and TuMV–Col-0 interactions, an increase up to the 7 dpi time point was observed. However, this observation did not take into account the subcellular distribution of glutathione in plant cell compartments, which could be important in response to TuMV.

**Figure 6:**
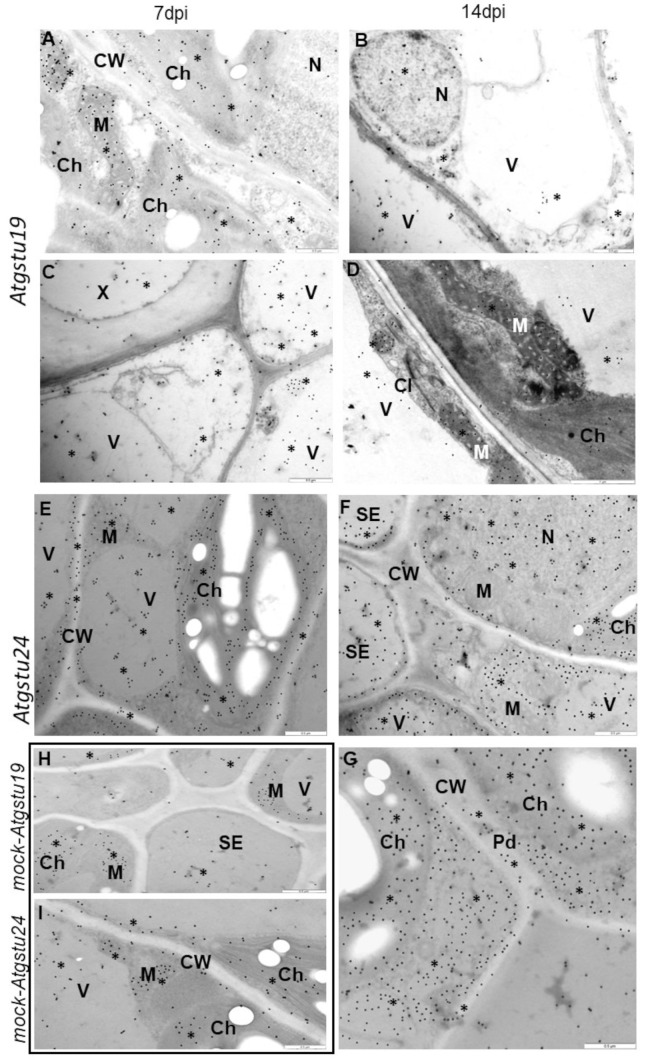
Immunogold labeling of glutathione deposition in *Atgstu19* (**A**–**D**) and *Atgstu24* (**E**–**G**) 7 dpi (**A**,**C**,**E**) and 14 dpi (**B**,**D**,**F**,**G**) after TuMV and mock-inoculation (in frame (**H**,**I**)) leaves. (**A**) Glutathione (*) deposition in chloroplast (Ch), mitochondria (M) and cytoplasm of mesophyll cells. Scale bar 0.5 μm. (**B**) Glutathione (*) in nucleus (N) and cytoplasm of mesophyll cell. Scale bar 0.5 μm. (**C**) Glutathione (*) in vacuole (V), cytoplasm and xylem tracheary element (X) in xylem tissue. Scale bar 0.5 μm. (**D**) Glutathione (*) deposition in mitochondria (M), chloroplast (Ch) and vacuole (V) in palisade mesophyll. Virus cytoplasmic inclusions (CI) present in mesophyll cell. Scale bar 1 μm. (**E**) Glutathione (*) deposition in chloroplast (Ch), cytoplasm, vacuole (V). Glutathione (*) deposited in cell wall (CW) of mesophyll cell. Scale bar 0.5 μm. (**F**) Glutathione (*) deposition in nucleus (N), chloroplast (Ch) and cytoplasm of phloem cells. Glutathione (*) deposition presented also inside phloem sieve element (SE). Scale bar 0.5 μm. (**G**) Glutathione (*) deposition in chloroplast (Ch), cytoplasm and cell wall (CW) also in plasmodesmata (Pd) 14 dpi after TuMV inoculation. Scale bar 0.5 μm. (**H**) Glutathione (*) deposition in mitochondria (M) and chloroplast (Ch) in phloem of mock-inoculated Atgstu19 leaf. Glutathione also inside sieve element (SE). V-vacuole. Scale bar 0.5 μm. (**I**) Glutathione (*) deposition in chloroplast (Ch), mitochondria (M) and vacuole (V) in mesophyll cell of mock-inoculated Atgstu24 leaf. CW—cell wall. Scale bar 0.5 μm.

**Figure 7:**
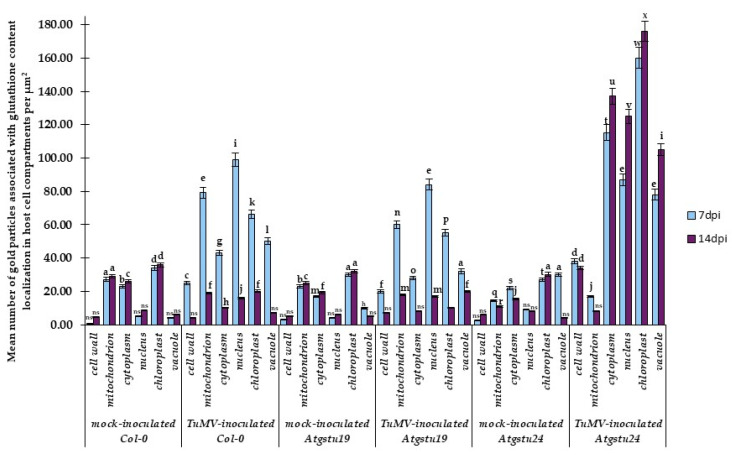
Quantitative immunogold labeling of glutathione content in mock- and TuMV-inoculated Col-0, *Atgstu19*, and *Atgstu24* leaves. The mean number of gold particles localized in specific compartments per µm2 at 7 and 14 dpi in mock- and virus-inoculated leaves is presented. Immunogold localization was validated using ANOVA. The mean values were calculated at *p* < 0.05 with post hoc Tukey’s HSD test. Statistically significant values are indicated by letters above the bars. Nonsignificant values are indicated as ns.

Therefore, immunogold localization (Figure 6A–I) with a validation step (Figure 7) was carried out to determine the glutathione content in cell compartments during interactions of TuMV with *Atgstu19*, *Atgstu24*, and Col-0. Based on the glutathione content analyses, 7 and 14 dpi time points were selected to present the glutathione distribution changes at the ultrastructural level. Subcellular distribution confirmed that TuMV inoculation induced a higher glutathione deposition between 7 and 14 dpi in plant cells, compared with mock-inoculated plants, but the most dynamic induction in the resistance-like reaction was observed during the TuMV–*Atgstu24* interaction (Figure 6E–G,I and Figure 7). However, in mock-inoculated *Atgstu24* plants, most frequent depositions in vacuoles and chloroplasts were observed at 7 dpi (Figure 6I and Figure 7), whereas in mock-*Atgstu19* plants, glutathione was mainly observed in chloroplasts and mitochondria (Figure 6H and Figure 7). During the susceptible TuMV–*Atgstu19* interaction, glutathione induction was noticed only up to the 7 dpi time point and depositions mainly occurred in the cell nucleus, mitochondria, and chloroplasts (Figure 6A,C,H and Figure 7). Moreover, glutathione deposition significantly decreased between 7 and 14 dpi in virus-inoculated *Atgstu19* and Col-0 plants (Figure 6B,D and Figure S1B(E’,F’)). Furthermore, when TuMV infection was fully developed at the 14 dpi time point, the glutathione level decreased and was statistically significant only in vacuoles and mitochondria (Figure 6B,D and Figure 7). Glutathione localization in mesophylls and vascular tissues of *Atgstu24* plants in resistance-like reaction to TuMV indicated the most frequent glutathione content deposition in chloroplasts, cytoplasm, and nucleus (Figure 6E–G). Moreover, the induction of glutathione in the cell wall at 7 dpi in the TuMV–Atgstu24 interaction was more dynamic than in the TuMV–*Atgstu19* interaction and mock-inoculated plants (Figure 6E–G and Figure 7). However, the level of deposition in mitochondria in TuMV–*Atgstu24* at 7 dpi was similar to that of mock-inoculated *Atgstu24* plants.

### 2.4. Relative Expression of Selected GSTU Genes in Virus-Inoculated Col-0, Atgstu19, and Atgstu24 Plants Correlated with Increased Susceptibility or Resistance-like Tendency

**Figure 8:**
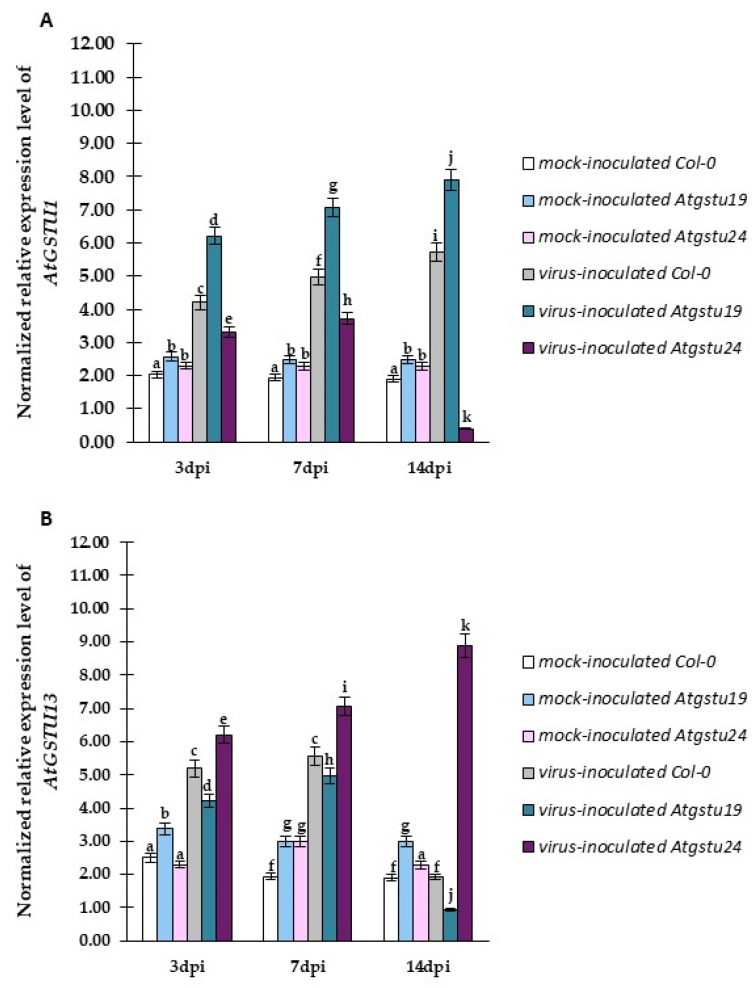
The normalized relative expression levels of *AtGSTU1* (**A**) and *AtGSTU13* (**B**) calculated based on the mean expression of *AtEf1α* and *AtF-Box* reference genes in mock- and virus-inoculated Col-0, *Atgstu19*, and *Atgstu24* plants between 3 and 14 dpi. The mean values of the normalized expression levels were calculated using ANOVA and Tukey’s HSD test at *p* < 0.05. Statistically significant values are indicated by letters above the bar.

**Figure 9:**
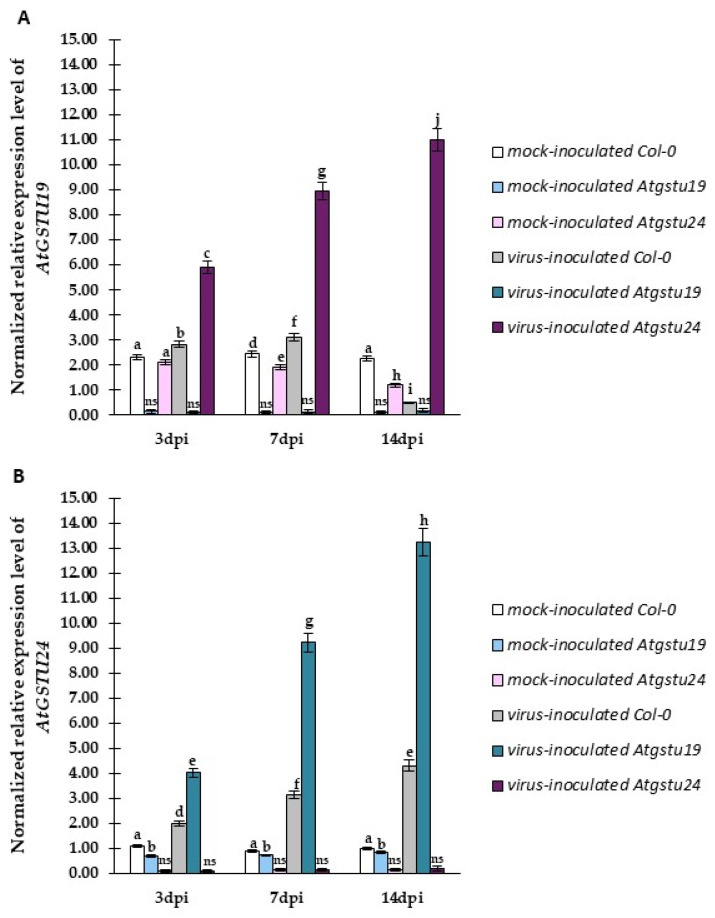
The normalized relative expression levels of *AtGSTU19* (**A**) and *AtGSTU24* (**B**) calculated based on the mean expression of AtEf1α and AtF-Box reference genes in mock- and virus-inoculated Col-0, Atgstu19, and Atgstu24 plants between 3 and 14 dpi. The mean values of the normalized expression levels were calculated using ANOVA and Tukey’s HSD test at *p* < 0.05. Statistically significant values are indicated by letters above the bar. Nonsignificant values are indicated as ns.

GST genes encode ubiquitous and multifunctional enzymatic proteins valid for the host plant and play a crucial role in the regulation of the concentration of glutathione under stress and normal conditions. The precise modulation of glutathione levels is important in the host’s response to viruses. Our findings based on TuMV concentration and significant changes and distribution in the glutathione content suggested important differences in response to TuMV between Col-0, *Atgstu19*, and *Atgstu24* plants. Therefore, the normalized relative expression tendency was evaluated using quantitative polymerase chain reaction (qPCR) analyses, described as the pathogen reaction related to *GSTU* genes, such as *AtGSTU1* and *AtGSTU13* [25,45] (Figure 8A,B). Moreover, taking into account the TuMV concentration in *Atgstu19* and *Atgstu24* mutants, the expression levels of *AtGSTU19* and *AtGSTU24* were tested in Col-0, *Atgstu19*, and *Atgstu24* plants as well (Figure 9A,B).

The normalized expression of the *AtGSTU1* gene was significantly higher than that of mock-inoculated plants and between 3 and 14 dpi in Col-0 (1.36-fold) and *Atgstu19* (1.27-fold) plants (Figure 8A). However, *AtGSTU1* expression was much higher in *Atgstu19* plants than in Col-0 plants, whereas, in *Atgstu24* plants, it was only induced between 3 and 7 days dpi (1.12-fold), and, compared with mock-inoculated plants (1.12-fold), it was drastically reduced between 7 and 14 dpi.

On the contrary, *AtGSTU13* expression was more significantly upregulated (1.43-fold) between 3 and 14 dpi, compared with mock-inoculated plants, and to the highest levels in the *Atgstu24*–TuMV interaction (Figure 8B). Moreover, *AtGSTU13* was downregulated at 7 dpi by 2.9-fold in Col-0 plants and 5.23-fold in *Atgstu19* plants in the susceptible interaction but was induced between 3 and 7 dpi, compared with mock-inoculated plants.

The analyses of *AtGSTU19* and *AtGSTU24* expression confirmed that *Atgstu19* and *Atgstu24* are both knockout mutant plants of the respective genes (Figure 9A,B). Moreover, it was also revealed that the *AtGSTU19* expression was highly upregulated in resistance-like reaction in the TuMV–*Atgstu24* interaction (1.86-fold) between 3 and 14 dpi. However, in susceptible Col-0 plants, *AtGSTU19* expression was upregulated only 1.1-fold between 3 and 7 dpi and highly downregulated (6.25-fold decrease). On the contrary, *Atgstu24* plants showed stable and high induction of the *AtGSTU19* gene during all analyzed time intervals (3.54-fold increase between 3 and 14 dpi). Moreover, the *AtGSTU19* gene was also slightly induced in Col-0 plants between 3 and 14 dpi.

The results of the analyses of TuMV concentration and normalized relative *AtGSTU* gene expression showed that the resistance-like reaction was associated with virus limitation in the TuMV–*Atgstu24* interaction and revealed the upregulation of *AtGSTU13* and *AtGSTU19* genes and the downregulation of *AtGSTU1* gene. However, virus propagation susceptibility observed in the TuMV–*Atgstu19* interaction was accompanied by the increased expression of *AtGSTU1* and *AtGSTU24* genes with the downregulation of *AtGSTU13*. Therefore, for an in-depth understanding of the relationship between *Atgstu19* and *Atgstu24* plants’ responses to TuMV inoculation, the correlation between the change of susceptibility/or resistance (based on TuMV concentration) and modulation of the *AtGSTU* gene expression (Tables S2–S5) was evaluated based on Pearson’s correlation coefficients (PCCs). The PCCs confirmed a high positive correlation between *AtGSTU13* (Table S3) or *AtGSTU19* (Table S4) expression and TuMV concentration, along with a high negative correlation between *AtGSTU1* (Table S2) expression changes and resistance-like reaction in the *Atgstu24*–TuMV interaction. Moreover, in susceptible Col-0 and susceptible *Atgstu19* plants, a positive correlation between the increased concentration of TuMV and increased *AtGSTU1* (Table S2) and *AtGSTU24* (Table S5) expression was observed. However, the values of PCCs were significantly higher in virus-inoculated *Atgstu19* plants than in virus-inoculated Col-0 plants. Furthermore, PCCs indicated the potential role of *AtGSTU13* and *AtGSTU19* genes with an increased expression level in the resistance-like reaction.

### 2.5. Significant Modulation of GST and GR Activity as Factors for Increased Susceptibility or Resistance-like Tendency in TuMV–Atgstu19 and TuMV–Atgstu24 Interactions

**Figure 10:**
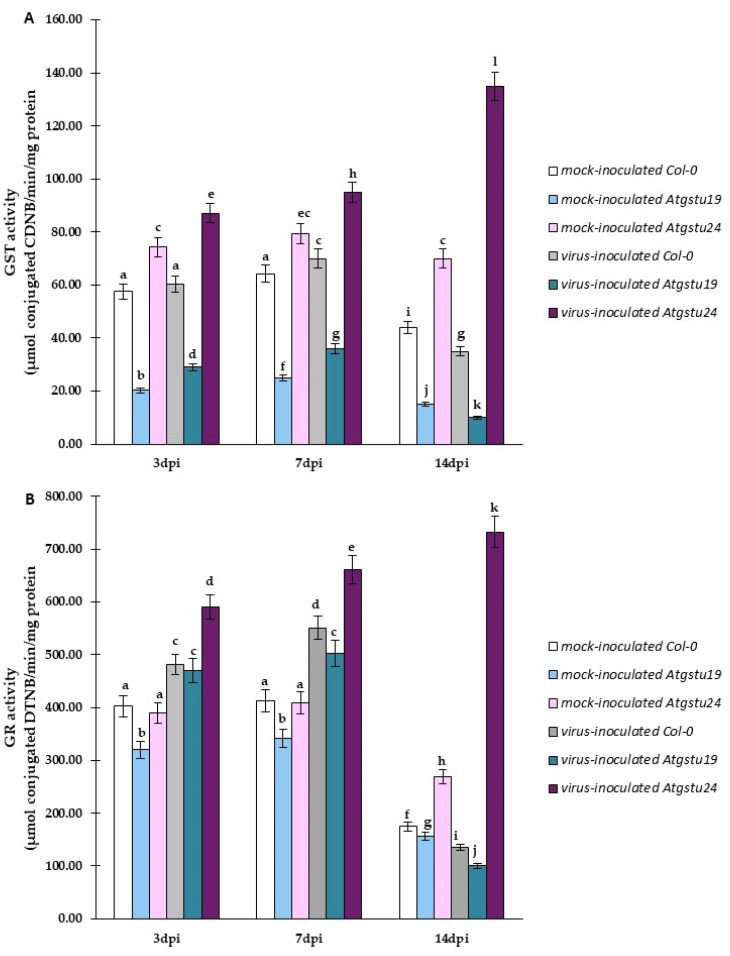
GST (**A**) (in nanomoles of conjugated CDNB) and GR (**B**) (in nanomoles of conjugated DTNB) activities in the leaves of TuMV- and mock-inoculated Col-0, *Atgstu19*, and *Atgstu24* plants between 3 and 14 dpi. The mean activities were calculated using ANOVA and Tukey’s HSD test at *p* < 0.05. Statistically significant values are indicated by letters above the bars.

The glutathione content and the modulation of *AtGSTU* genes suggested the important role of GSTUs in the modulation of different host responses to TuMV inoculation. Therefore, to better understand different responses to virus infection, the enzymatic activity of GST (EC 2.5.1.18) was evaluated, which is considered the marker for resistant/or even hypersensitive reaction to viral pathogens [16] and GR (EC 1.8.1.7) enzymatic activity, which is one of the most important enzymes in restoring GSH using GSSG during oxidative protection. GST and GR activities were significantly increased in TuMV-inoculated *Atgstu24* (resistance-like reaction) plants from 3 to 14 dpi, 1.55-fold and 1.2-fold, respectively (Figure 10A,B). In contrast, in TuMV-inoculated Col-0 and *Atgstu19* (susceptibility) plants, they were induced only between 3 and 7 dpi, following which they significantly decreased at the 14 dpi time point (Figure 10A,B). Moreover, GST and GR activities were, in general, lower in virus-inoculated Atgstu19 plants than in TuMV-inoculated Col-0 plants. Furthermore, reduction in both activities after the 7 dpi time point was also more severe in the TuMV–*Atgstu19* interaction. This relationship suggested that the modulation of GR and GST activities also strongly correlated with *AtGSTU* gene expression and the content of both glutathione forms, which are critically dependent on *A. thaliana* plants’ response to TuMV inoculation—susceptibility or resistance-like reaction.

## 3. Discussion

This study explored for the first time the response of *Atgstu19* and *Atgstu24* mutants to TuMV infection. The findings of this study showed significant differences in *AtGSTU* gene expression, virus concentration, ultrastructural alterations, glutathione content, and glutathione transferase and reductase activities during TuMV–*Atgstu19* and TuMV–*Atgstu24* interactions, compared with Col-0 (wild-type) and mock-inoculated plants. Moreover, *A. thaliana* plants with a knockout of single GST from the tau group showed different responses to TuMV inoculation. In general, plant GSTs are postulated as enzymes involved in diverse functions, ranging from plant detoxification and ROS homeostasis to signaling molecules and adaptors in biotic stress [46,47,48]. Arabidopsis genome harbors 54 GST genes grouped into seven classes. The largest two classes—GSTF and GSTU—are specific for plants that display high inducibility by biotic factors [24,49]. Our previous studies on PVY infection indicated that potato GST from the *phi* class *StGSTF* genes is differentially regulated during interactions between *S. tuberosum* cultivars and PVY^NTN^ [16]. We confirmed that the enhanced expression of *StGSTF2* corresponded to HR induction and reduced PVY^NTN^ concentration. Moreover, as reported in previous studies, StGSTF2 participated not only in resistance response but also in systemic infection in susceptible potato reaction. Therefore, we decided to examine whether the second largest GST class—*tau*—has an impact on plant–TuMV interactions. In Arabidopsis, the GSTU class includes 28 members [38]. Some studies have described the role of GSTUs in detoxification (abiotic stress) [50]. It is reported that plants overexpressing *GSTU* genes show enhanced stress resistance [31]. Moreover, GSTU members are involved in response to different abiotic stress factors and are well studied [37,51]. However, knowledge about GSTU functions in plant–pathogen interactions is still far from adequate. In our experiments, *A. thaliana gstu19* knockout mutants exhibited susceptible response to TuMV, which was confirmed by the induction of *TuMV-CP* expression, a higher TuMV concentration between 3 and 14 dpi, ultrastructural alterations with virus particles, and virus cytoplasmic inclusion depositions. Moreover, among the selected *AtGSTU* genes, *AtGSTU1* and *AtGSTU24* were highly induced between 3 and 14 dpi during TuMV infection, compared with not only mock-inoculated plants but also wild-type susceptible Col-0. Furthermore, in our studies *AtGSTU13* was activated in a susceptible reaction only up to 7 dpi until symptoms appeared (7 dpi time point); therefore, it can be potentially possible that GSTU13 contributed in symptoms development in *Agstu19*-TuMV pathosystem. Moreover, *AtGSTU13* was also dynamically activated (between 3 to 14 dpi) in an *Atgstu24*-TuMV resistance-like reaction. Furthermore, PCCs analyses indicated the potential role of AtGSTU13 with an increased expression level in the resistance-like reaction, when AtGSTU13 correlated with the reduction in virus concentration, in contrast to the susceptible reaction in *Atgstu19*. Therefore, AtGSTU13 can potentially act a dual role, contributing in resistance reaction, as well as a participant in symptoms development in susceptible reaction. These findings on AtGSTU13 are partially consistent with those of the report presented by Zhang and co-authors [52], in which glycine max GSTU13 was associated with the development of symptoms induced by *Soybean mosaic virus* (SMV) at both transcriptional and protein levels.

Moreover, a strong correlation between TuMV concentration and *AtGSTU1* and *AtGSTU24* expression was observed between 3 and 7 dpi in the susceptible TuMV–Atgstu19 interaction. Therefore, it can be postulated that AtGSTU1 and AtGSTU24 contribute to the susceptibility of *A. thaliana* to TuMV and the limitation of oxidative stress, whereas, similar to StGSTF2 in PVY^NTN^–potato interactions, AtGSTU13 is involved in the induction of virus symptoms in susceptible reactions.

Interestingly, transcriptomic analyses revealed that the GST expression profile can be differentially regulated in plant–virus interactions, such as pepper leaves infected with *Obuda pepper virus* [53], *Rice stripe virus* disease in Arabidopsis [54], *Beta vulgaris*–*Beet necrotic yellow vein virus* (BNYVV) interactions [55], and response of watermelon to *Cucumber green mottle mosaic virus* [56]. Moreover, in the response of susceptible *A. thaliana* to *Cauliflower mosaic virus*, systemic induction of GST1 with increased virus titers and development of symptoms was observed [57]. Furthermore, Pavan Kumar et al. [58] observed the accumulation of GST proteins in systemically infected leaves in soybeans susceptible to *Mungbean yellow mosaic India virus* and *Mungbean yellow mosaic virus*. In addition, Skopelitou et al. [19] reported that GSTU10-10 in soybean was specifically upregulated after systemic infection induction caused by SMV. In addition, Chen et al. [59] observed that NbGSTU4 was upregulated in the *Bamboo mosaic virus*–*Nicotiana benthamiana* interaction, confirming the ability of binding to the UTR region of (+) s virus RNA, leading to effective replication. The protein–protein interaction between host plants and pathogens is an active area of research that can not only widen our understanding of virus–plant interaction but also facilitate resistance breeding [60]. The ability of virus interaction with host proteins in *Potyviridae* family is a frequent feature during induction and support of virus infection in different host reaction [59,60]. In this context, it could not be excluded that GSTU19 or GSTU24 proteins could be potentially involved in a form of interaction with virus, virus proteins, or other host proteins, but this fact must be separately confirmed in further studies. Dean et al. [61] reported that in *N. benthamiana*–*Colletotrichum destructivum* and *N. benthamiana*–*Colletotrichum orbiculare* interactions, two *GSTU* genes were highly induced: *NbGSTU1* and *NBGSTU3*. When the transcription of *NbGSTU1* was blocked by gene silencing, the resistance to *C. orbiculare* was highly suppressed. Moreover, a 67% higher fungi colonization and a 130% higher number of lesions caused by *C. orbiculare* were observed, compared with mock-inoculated plants. Moreover, in *A. thaliana*, a *tau* class gene *GSTU13* was identified as an indispensable component of the immune pathway, as the lack of functional GSTU13 resulted in enhanced susceptibility to the following fungal pathogens: *Plectosphaerella cucumerina*, *Colletotrichum*, or *Erysiphe* [33]. Similarly, dysfunction in GSTU19 induced a susceptible interaction with a virus, even more highly dynamic than in susceptible Col-0 plants. The findings of this study and those previously mentioned showed that even individual *GSTU* genes may suppress the resistance of plants to pathogens. Therefore, we postulated that GSTU19 in the *A. thaliana*–TuMV interaction can act as a crucial factor for the resistance of plants to TuMV.

Quite a different scenario was observed in the *Atgstu24*–TuMV pathosystem. In *Atgstu24* mutants, virus titers quantified by DAS-ELISA were lower between 3 and 14 dpi, which was confirmed by the decrease in the relative expression of *TuMV-CP*, especially between 7 and 14 dpi, compared with Col-0 and *Atgstu19*. Moreover, ultrastructural analyses with virus capsid immunogold labeling indicated that in the TuMV–*Atgstu24* interaction, virus particles were observed in lower frequency and only in vacuoles. Furthermore, in these interactions, neither the induction of virus cytoplasmic inclusion typical for Potyvirus susceptible reaction nor chloroplast lamella disorganization [43] was observed. On the contrary, in the *Atgstu24*–TuMV pathosystem, higher levels of virus limitation and resistance-like reaction were observed, compared with wild-type reaction and, in particular, *Atgstu19*. This was accompanied by a significant reduction in TuMV-CP deposition between 7 and 14 dpi. In the present study, relative expression analyses of the selected *AtGSTU* genes indicated that *AtGSTU13* and *AtGSTU19* were highly induced between 3 and 14 dpi. Moreover, the dynamic increase in *AtGSTU19* and *AtGSTU13* expression strongly correlated with the reduction in virus concentration, in contrast to the susceptible reaction in *Atgstu19*. Furthermore, in *Atgstu24* response to TuMV, *GSTU1* was also induced, only up to the time point when infection symptoms appeared at 7 dpi, whereas it was highly reduced between 7 and 14 dpi. We postulated that in the response of *Atgstu24* to TuMV, GSTU19 and GSTU13 contribute to resistance-like reaction induction, whereas GSTU1 contributes to symptom development. The induction of *AtGSTU19* and *AtGSTU13* in Arabidopsis response to TuMV was similar to the activation of *StGSTF2* in the hypersensitive response of potato to PVY^NTN^, which correlated with *PVY-CP* downregulation [16]. As reported by Fodor et al. [62], GST in general can play a pivotal role in controlling resistance-like HR. Moreover, increased expression of *NtGSTU1* was observed between 3 and 6 h after virus inoculation, causing a reduction in TMV replication [63]. Furthermore, the expression of GST genes was significantly activated in the accumulation of BNYVV resistance [64]. As observed by Rodriguez-Pena et al. [65], GSTU4 downregulation caused a significant reduction in the accumulation of *Barley mosaic virus* and *Potato virus X* but had no influence on *Cucumber mosaic virus*. Satoh et al. [66] postulated that in resistant plants, almost all induced GST genes were expressed to higher levels in response to *Rice tungro spherical virus* of rice cultivars. Wang et al. [67] reported on a virus-induced gene silencing system, indicating that GSTU6 in *Triticum aestivum* has an important role in resistance to Bgt (*Blumeria graminis* f. sp. *tritici*) but not to Pst (*Puccinia stitiformis* f. sp. *tritici*). The overexpression of *TaGSTU6* in Arabidopsis induced resistance to *Pseudomonas syringae* pv. Tomato DC3000 [67]. In addition, many GSTs of the GSTU class were strongly activated in the leaves of *A. thaliana* during *Alternaria brassicicola* infection [68]. *GSTU1*, *GSTU11*, and *GSTU10* genes were actively induced two days after fungus inoculation. Glutathione transferases are known for their role in maintaining the physiological redox state of the plant cell [21]. Several studies reported that the expression and activity of GST are affected by the GSH content and GSH/GSSG ratio [69]. Therefore, we also examined glutathione distribution and concentration with GST and GR activities in TuMV–*Atgstu19* and TuMV–*Atgstu24* interactions. Numerous studies have shown that the induction of the glutathione content is important for plant disease resistance response. For example, Király et al. [63] demonstrated that TMV resistance correlated with the increase in glutathione and Cys levels. Moreover, treatment of tobacco plant leaf disk with the cysteine precursor OTC resulted in glutathione accumulation and a significant reduction in the TMV content, as reported by Güllner et al. [17]. The findings of the present study demonstrated a resistance-like reaction in the *Atgstu24*–TuMV interaction: the highly dynamic increase in the glutathione form between 1 and 14 dpi, accompanied by a significant increase in the GSSG form. Moreover, the levels of GSH and GSSG were the highest in *Atgstu24*–TuMV interactions, compared with *Atgstu19*–TuMV and Col-0–TuMV interactions. These observations were quite different from those presented by Horváth et al. [50], where the oxidized glutathione form was not significantly different between the *Atgstu19* knockdown line and *Atgstu24* knockout plants under salt stress conditions. However, plants that underwent *Atgstu24* knockout mutant salt treatment showed an increased content of total glutathione and a high GSH/GSSG ratio. A similar tendency to *Atgstu24*–TuMV interactions was observed in PVY^NTN^–resistant potato Neptun [16]. Higher concentrations of GSH and GSSG concentrations were observed in PVY^NTN^ inoculation between 1 and 21 dpi, compared with susceptible reactions and mock-inoculated plants. These findings are consistent with those of Singh et al. [70], who reported that the GSSG concentration was higher in resistant plants, compared with susceptible cultivars. Moreover, as reported by Király et al. [71] and Künstler et al. [72], a high GSSG content indicates the important role of glutathione in oxidative stress in TMV resistance reaction, suggesting the suppression of defense response. Furthermore, different stress conditions usually change the glutathione concentration and shift the glutathione ratio toward the GSSG form [12,73].

Significant differences in the glutathione concentration were observed in *Atgstu19*–TuMV and Col-0–TuMV interactions. Moreover, these observations are consistent with the data obtained in susceptible PVY^NTN^–potato Irys reaction [16]. During *Atgstu19*–TuMV interaction, the GSH content was highly induced up to 7 dpi, whereas the GSSG pool was only induced at the 1 dpi time point. Moreover, in susceptible *Atgstu19*–TuMV interactions, the total glutathione content (GSH + GSSG) increased up to 3 dpi, whereas it increased only between 3 and 7 dpi in Col-0 plants. Importantly, the decrease in the GSSG form started earlier (at 3 dpi) and was more dynamic than in Col-0. Finally, in susceptible *Atgstu19*–TuMV and Col-0, the GSH and the total glutathione content dynamically decreased at the 7 dpi time point, especially in *Atgstu19*–TuMV to a level even lower than in mock-inoculated wild-type Col-0. Considering the dynamic nature of the glutathione content during Col-0 and Atgstu19 induction in response to virus inoculation, TuMV-CP expression induction, and the tendency of virus titers, it can be concluded that the *Atgstu19*–TuMV interaction was more enhanced for susceptibility reaction than in Col-0 plants. This was also confirmed by ultrastructural alterations visualized by transmission electron microscopy and TuMV-CP deposition between *Atgstu19*–TuMV and Col-0 interactions [44]. A similar tendency of the decrease in the glutathione content in susceptible interactions after the development of systemic symptoms was noticed by Hakmaoni et al. [74], where the GSH content decreased in *N. benthamiana* susceptible to pepper mild mottle virus. It can be assumed that in line with Hernandez et al. [75], glutathione fails to efficiently detoxify ROS in susceptible response and to prevent the development of systemic symptoms induced by the virus.

The ultrastructural analyses of glutathione localization in inoculated *A. thaliana* leaf tissues also revealed significant differences in the deposition between mock- and virus-inoculated plants and between *Atgstu19* and *Atgstu24* interactions. In mock-inoculated Col-0 plants, the most intense GSH deposition was detected in the following order: chloroplasts, mitochondria, and cytoplasm, similar to *Atgstu19*-mock-inoculated plants. In *Atgstu24*-mock plants as well, the deposition was the highest in chloroplasts, but quantification of gold granules indicated the highest GSH deposition in vacuoles, followed by chloroplasts. In general, gold labeling confirmed the glutathione content tendency in response to the virus in all interactions observed by HPLC analyses. Therefore, in *Atgstu24* interactions, a steady increase in GSH localization was observed. On the contrary, in Col-0 and *Atgstu19* interactions, localization was induced only up to 7 dpi, whereas an active decrease in localization was observed between 7 and 14 dpi, compared to mock-inoculated and to *Atgstu24*. Indeed, the highest levels of glutathione localization were reported in *Atgstu24*–TuMV. In *Atgstu24*–TuMV, the highest deposition was observed in the chloroplasts (much higher than in mock-inoculated *Atgstu24*), followed by the cytoplasm and the nucleus. The increase in the glutathione content in chloroplasts is an important factor for controlling ROS and symptom development. The findings of this study are consistent with those presented by Zechmann [76], where plants resistant to TMV showed the most intense glutathione localization in chloroplasts. These findings also agree with those of Clemento-Moreno et al. [77], who presented a strong increase in the glutathione content in chloroplasts during the susceptible response to *Plum pox virus*. Moreover, individual genes from *Plum pox virus* and TuMV expressed in plants can repress or change the virulence of virus to plants [60]. Because of this fact, host resistance or susceptibility to the virus is the outcome of clash between the plant host and invading virus. Garcia-Ruiz [77] postulated that in case of Potyviruses we have pro-viral and anti-viral host factors co-opted by virus. Yang et al. [60] and Garcia-Ruiz et al. [78] suggested that main process that regulates TuMV infection and susceptible/resistant reaction of host cell (such as RNA-silencing) are associated with Dicer-like (DCL2, DCL4), RNA-dependent RNA polymerase (RDR1, RDR6), and ARGONAUTES (AGO1, AGO2 and AGO10), which are located and active in plant nucleus or cytoplasm. In this context, the appropriate levels of glutathione and ROS (needed for precise targeted resistance response) in particular parts of cells could be important for signal transduction and optimal functioning of antiviral host factors. Therefore, the decreased levels of glutathione observed in susceptible *Atgstu19* plants could disturb the natural defense process located in specific cell regions and promote viral infection. Moreover, in the *Atgstu24*–TuMV interaction, a highly induced GSH localization at the 7 dpi time point and a steady increase between 7 and 14 dpi in all cell compartments, except for mitochondria, were observed when the GSH content significantly decreased. Király et al. [63] and Simon et al. [79] reported similar glutathione depletions in mitochondria in correlation with the hypersensitive response to TMV or *Botrytis cinerea*. Moreover, the lower level of glutathione in mitochondria may lead to its dysfunction and activation of the resistance response to the pathogen. Furthermore, only in the resistance-like reaction in *Atgstu24*–TuMV was a statistically steady stable significant cell wall deposition (7–14 dpi) observed. However, in virus-inoculated Col-0 and *Atgstu19*, cell wall localization was detected, but at the 7 dpi time point, it decreased to a nonsignificant level. As previously postulated by Tolin et al. [80] and Vanacker [81], the GSH content in apoplasts is an important factor in sensing and signaling stress and, when the apoplast pool is more oxidized, can also play a role in adaptation to biotic stress. It can be concluded that only in a resistance-like reaction did the glutathione cell wall pool show a significant virus response, but further studies are needed to elaborate on this tendency. In contrast, in susceptible response to TuMV in *Atgstu19* and Col-0 inoculated tissues, the highest glutathione induction at 7 dpi was observed in the following order: nucleus, mitochondria, chloroplasts, and vacuoles. After symptom development between 7 and 14 dpi, a significant reduction in localization was noticed in all cell compartments in both interactions. The tendency of glutathione localization in the nucleus and the cytoplasm is quite similar to data reported in PVY^NTN^–potato interactions [16]. Glutathione exchange was observed in mock- and virus-inoculated tissues. In several plant–pathogen interactions, the induction of the GSH content in the nucleus and activation of the whole glutathione pool can also stimulate diffusion into the nucleus after cytoplasm synthesis [82,83,84]. Moreover, the deposition of glutathione in the nucleus may lead to plant defense through an environment that reduces antioxidant enzymes.

In our experiments, several *GSTU* genes were differentially expressed in *Atgstu19*–TuMV and *Atgstu24*–TuMV pathosystems, which was followed by significantly differentially changed glutathione reduced and oxidized forms and was accompanied by changes in GST and GR activities. Gullner et al. [85] and Wu et al. [86] studied the GST activity induced by the virus in sorghum cultivars’ interaction with *Sugarcane mosaic virus* (ScMV). They observed a more than 50% increase in the GST activity in the first 3 dpi in ScMV-resistant response, whereas susceptible cultivars exhibited a decreased GST activity. Moreover, they postulated that the GST activity may be associated with the resistance response to the virus. An increase or decrease in the GST activity can be a marker of susceptibility. Therefore, strong induction in *Atgstu24*–TuMV interaction 3–14 dpi was correlated with the resistance-like reaction, whereas weaker induction only between 3 and 7 dpi and a decrease between 7 and 14 dpi were associated with susceptibility. Moreover, *Atgstu19* knockout line–TuMV interaction revealed a lower GST activity than Col-0-virus-inoculated and Col-0-mock inoculated plants. Col-0 exhibited a less susceptible reaction than *Atgstu19* to TuMV. Horváth et al. [37] reported a closely related tendency in *Atgstu19* knockdown plants treated with salt stress. Similarly, a long-term systemic infection caused by *Plum pox virus* revealed a strong decrease in the GST and GR activities [87]. In addition, the GR activity also showed the same tendency as the GST activity in susceptible reactions with Col-0 and *Atgstu19*. Virus-inoculated Col-0 plants revealed a higher GST activity than in *Atgstu19*–TuMV interaction. However, in *Atgstu19*–TuMV, a lower activity was observed than in the resistance-like reaction in Atgstu24–TuMV. Moreover, the highest activity was reported in *Atgstu24*–TuMV between 3 and 14 dpi, compared with mock- and all virus-inoculated plants. Fodor et al. [62] reported a significant decrease in the GR activity in the inoculated lower tobacco leaves 2 days after TMV inoculation, whereas, at the 3 dpi time point, it had already increased reaching 175% of control 7 days after inoculation. On the contrary, the TMV infection of lower leaves significantly induced the GR activity in the upper leaves 12 days after inoculation (160% of control). Clarke et al. [88] reported a 6.7-fold decrease in the GR activity in susceptible reaction in *White clover mosaic virus* (WCMV)–bean interactions 10 dpi virus inoculation. Li and Burrit [89] reported a 48% decrease in the GR activity in *Dactylis glomerata*–*Cocksfoot mottle virus* (CfMV) interaction between 3 and 5 dpi. Amari et al. [90] reported a 21% decrease in the GR activity in *Prunus necrotic ringspot virus* (PNRSV)–apricot interaction. The pool of reduced glutathione GSH may be fueled by the GR activity. GR catalyzes the reduction of glutathione disulfide (GSSG) to two molecules of GSH, and thus electron transfer from NADPH and is crucial for maintaining the glutathione redox potential in different plant cell compartments.

## 4. Materials and Methods

### 4.1. Plant Material, Virus Inoculation, and DAS-ELISA and Molecular Test for TuMV Levels

Changes occurred during viral infections induced by TuMV and related to glutathione metabolism. *Arabidopsis thaliana* (L.) Heynh wild-type (Col-0) plants and *A. thaliana* lines containing a T-DNA insertion were used: in *AtGSTU24* (*At1g17170*, line SALK_034472) compensation of mutation in Arabidopsis glutathione transferase (*AtGSTU*) genes under control or salt stress conditions and in *AtGSTU19* (*AT1G78380.1*, line NASC WiscDsLox430F05). *Athgstu24* are knockout mutants, as reported by Horváth et al. [37]; according to the results presented in this paper, *Atgstu19* is also a knockout mutant. All homozygous mutant seeds were kindly provided by Jolán Csiszár Laboratory. For TuMV inoculation described below, 18-day-old plants without any lesions and/or alterations were used. Healthy and mock-inoculated *Athgstu24* and *Athgstu19* revealed phenotype differences (Figure S1A).

*Arabidopsis thaliana* Col-0 and mutant plants were mechanically inoculated following the procedure of Otulak-Kozieł et al. [44], Tomilson [91], and Walsh and Jenner [92] using the TuMV inoculum (isolate PV-0104 was kindly provided by Leibniz Institute, Braunschweig, Germany) in phosphate buffer [93]. Interestingly, at the 7 dpi time point, more severe TuMV symptoms were observed in *Atgstu19* andCol-0 than in *Atgstu24* (Figure S1A). Leaves of mock- and TuMV-inoculated plants were assessed for the presence of the virus using DAS-ELISA and qPCR. DAS-ELISA was performed with the primary antibodies against the TuMV (Bioreba, Reinach, Switzerland, catalog number: 161012), following the procedure of Otulak-Kozieł et al. [44]. Each repeat was performed in a new ELISA plate with samples. For each test, samples from 25 mock-inoculated or TuMV wild-type or mutant plants were combined separately; the same was carried out for TuMV-inoculated plants. All DAS-ELISA tests were performed using the same reagents. The readings of OD_405 nm_ values were acquired after 60 min in duplicate, 3, 7, and 14 dpi. The mean OD_405 nm_ values were statistically assessed by a one-factor analysis of variance (ANOVA) using the Statistica software (version 13.0; StatSoft and TIBCO Software Inc., Palo Alto, CA, USA), as described by Kozieł et al. [94]. For a more precise assessment, the corrected mean OD_405 nm_ values were computed as presented in a previous study [94] and used to compare the relative level of virus presence/concentration in plants. The cutoff point was also calculated by using the formula suggested by Bioreba (Reinach, Switzerland) [95] and presented previously by Otulak-Kozieł et al. [44].

This calculated cutoff point was 0.1412. The readings of OD_405 nm_ were compared to the calculated cutoff point, and all OD_405 nm_ values greater than 0.1412 were considered positive (confirmed presence of virus) [96]. Significant threshold/cutoff point values of DAS-ELISA confirmed the presence of the virus in all inoculated *A. thaliana* plants. Moreover, to double-check the level of TuMV, qPCR of the *TuMV-CP* gene fragment was performed using the primers presented by Arous et al. [97], and the expression was compared with the mean expression of the plant host reference genes *AtEf1α* (*A. thaliana* elongation factor-1 alpha, Arabidopsis Information Resource (TAIR): At5g60390) and *AtF-Box* (*A. thaliana* F-box family protein gene, Arabidopsis Information Resource (TAIR): At5g15710), as presented by Lilly et al. [98]. The level of the virus is presented as the normalized expression of the *TuMV-CP* gene. For DAS-ELISA, *TuMV-CP* expression, and other analyses (microscopy, HPLC, GST/GR enzymatic activity), 50 plants were used (25 virus-inoculated and 25 mock-inoculated of each Col-0 and two glutathione transferase mutants). The analyses were performed in triplicate using a new set of plants every time.

### 4.2. Isolation of RNA and Genomic DNA (gDNA) for Selected Gstu Genes in TuMV-Infected Col-0, Atgstu19, and Atgstu24 Plants

To estimate the expression of *A. thaliana GSTU* genes in the plant host, molecular analyses were performed on the samples collected at 3, 7, and 14 dpi. Briefly, leaf samples (0.1 g of each sample) were collected from 25 mock- (buffer) or virus-infected plants of different types of Col-0 and mutant plants. RNA isolation, purification, and quality analyses were carried out following previously described procedures [16,99,100]. In addition, the absence of RNA contamination was verified by performing reverse transcription PCR using *AtEf1α* (*A. thaliana* elongation factor-1 alpha) and *AtF-Box* (*A. thaliana* F-Box protein family) as reference standards [98], which confirmed the absence of contaminating gDNA. Then, cDNA was synthesized using the NG dART RT Kit (EURx Sp. z o.o., Gdansk, Poland) as per the manufacturer’s instructions. Reverse transcription reactions were performed in a 10 µL volume using 1000 ng of RNA.

### 4.3. Analysis of Expression of Selected GSTU Genes in TuMV-Infected Col-0, Atgstu19, and Atgstu24 Plants Using qPCR

A real-time qPCR was performed using the Bio-Rad CFX96TouchTM apparatus (Bio-Rad Poland Sp. z o.o., Warsaw, Poland) and Fast SG qPCR Master Mix (2×) (EURx Sp. z o.o., Gdansk, Poland) for *AtEf1α* and *AtF-Box* reference genes. All qPCR tests were calibrated using previously prepared 5-point calibration curves (based on cDNA and gDNA). The following genes were selected based on pathogen reaction involvement [25,33,44] analyzed in qPCR: *A. thaliana GSTU1* (*AtGSTU1*, AT2G29490) and *GSTU13* (*AtGSTU13*, AT1G27130). In addition, *GSTU19* (*AtGSTU19*, AT1G78380) and *GSTU24* (*AtGSTU24*, AT1G17170) were analyzed using qPCR expression. These host genes encoded protein products that were associated with the utilization of glutathione in response to stress (GSTU) [37]. The expression of the abovementioned GSTUs in *A. thaliana* was analyzed, and complete sequences were acquired from the TAIR database [101]. Moreover, gene expression was investigated in Col-0 and mutant plants using *AtEf1α* and *AtF-Box* as reference standards, as previously described [98]. The primers were acquired from previously published papers [37,97]. All the primers used in the experiments are presented in Table S6. The starting cDNA solution (used for generating calibration curves) was a fourfold-diluted mix of 12 randomly selected cDNA mixes. An eightfold-diluted cDNA mix was used to construct the calibration curve for gDNA. The subsequent calibration points were measured at fourfold dilutions in a 15 µL volume. A 5 µL solution of eightfold-diluted cDNA mix was added to the reaction mixture. The conditions used for qPCR analyses are presented in Table S7. The qPCR analyses were performed on 50 plants (25 virus-inoculated and 25 mock-inoculated of each Col-0 and two GST mutants) in triplicate using a new set of plants every time. Moreover, based on data from the expression of *AtGSTU1, AtGSTU13, AtGSTU19, and AtGSTU24* and concentration of TuMV (based on the expression of *TuMV-CP*), correlation analyses were performed. To compare/check the likelihood between the expression of different *GSTU* genes and the level of the virus, PCCs were estimated according to Wu et al. [102] and Manders et al. [103] by using Excel 2019 software (Microsoft, Poland, Warsaw). The pairwise correlations between *GSTU* gene changes and levels of TuMV were estimated at 3, 7, and 14 dpi in Col-0 and mutant plants. The results were presented in the form of a heat map generated using PCC values, and values higher than 0.68 were considered to reflect the strong positive correlation between analyzed pairs.

### 4.4. HPLC Analysis of Reduced (GSH) and Oxidized (GSSG) Forms and Total Glutathione Content

The GSH and GSSG contents in mock- and TuMV-inoculated Col-0, *Atgstu19*, and *Atgstu24* plants were measured by reversed-phase HPLC with fluorescence detection, as reported by Kranner [104], using the exact procedure presented by Otulak-Kozieł et al. [16]. They were estimated using the results of standards and presented as nmol g−1 FW (fresh weight). In HPLC analyses, 50 plants were used (25 virus-inoculated and 25 mock-inoculated of each Col-0 and two GST mutants). All analyses were performed in triplicate using a new set of plants every time.

### 4.5. Validation of GST and GR Activities in Leaves of TuMV-Infected Col-0, Atgstu19, and Atgstu24 Plants

To validate the GST and GR activity, Col-0, *Atgstu19*, and *Atgstu24* leaves were collected at the 3, 7, and 14 dpi time points after the inoculation of mock or TuMV. The GST activity was validated as described by Islam et al. [105] and Otulak-Kozieł [16], and the GST activity was determined based on its ability to conjugate GSH and 1-chloro-2,4-dinitrobenzene (CDNB) at 344 nm [106]. The results of the GST activity were presented as nanomoles of CDNB conjugated/min/mg total protein. The GR activity was determined by measuring the absorbance increment at 412 nm when 5,5′-dithio-bis(2-nitrobenzoic acid) (DTNB) was reduced by GSH, generated from GSSG, as proposed by Bela et al. [107]. The GR activity was calculated as the amount of reduced DTNB, in nanomoles of DTNB conjugated/min/mg total protein, ε420 = 13.6 mM^−1^ cm^−1^. To validate the enzymatic activity, 50 plants (25 virus-inoculated and 25 mock-inoculated of each Col-0 and two glutathione transferase mutants) were used. All analyses were performed in triplicate using a new group of plants every time.

### 4.6. Ultrastructural Analyses, Immunogold Localization of TuMV, and Glutathione Content Changes in TuMV-Infected Col-0, Atgstu19, and Atgstu24 Plants

To analyze the virus concentration using microscopic studies, the leaf samples of mock- and virus-inoculated potato plants at the 7 and 14 dpi time point were embedded and treated following the procedure of Zechmann et al. [82] and Kolb et al. [108] to assess the changes in the glutathione content. For ultrastructural analyses and immunolocalization of TuMV, the procedure reported by Kozieł et al. [109] was followed. Then, the leaf sections were mounted on Formvar-coated nickel grids, and immunogold localization was carried out, as described by Zechmann et al. [82] and Kozieł et al. [109], for glutathione and TuMV localization, respectively. The sections were counterstained with 2% uranyl acetate for 5 min and washed 5× for 2 min each with distilled water. To determine the localization of the glutathione content, primary polyclonal rabbit antibodies targeting the total glutathione content (in 1:100 dilution; Merck, Warsaw, Poland, catalog number: AB5010) and visualizing secondary antirabbit antibodies conjugated with 18 nm nanogold particles (Jackson ImmunoResearch Europe Ltd., Cambridgeshire, UK, catalog number: 711-215-152) were used. To analyze the localization of TuMV, primary polyclonal rabbit antibodies targeting TuMV (Bioreba, Reinach, Switzerland, catalog number: 161012) and visualizing secondary antirabbit antibodies conjugated with 18 nm nanogold particles (Jackson ImmunoResearch Europe Ltd., Cambridgeshire, UK; catalog number: 711-215-152) were used. The labeling specificity was determined by incubating the grids with the samples obtained from mock-inoculated plants and omitting the primary antibodies from the incubating solution. The immunogold-labeled sections on the grids were examined using a transmission electron microscope [110]. Then, protein labeling was quantified following the method of Luschin-Ebengreuth and Zechmann [111] in specific cell sections in the case of glutathione and globally in the case of TuMV. Statistical analyses were performed, as described by Otulak-Kozieł et al. [110]. The concentrations of gold particles in specific cell sections and globally were validated using ANOVA and post hoc Tukey’s HSD (honestly significant difference) test using Statistica software (version 13.0; StatSoft and TIBCO Software Inc., Palo Alto, CA, USA). ANOVA was used to estimate gold labeling. For the statistical estimation of immunogold labeling, infected and mock-inoculated materials were compared at the 7 and 14 dpi time point. The number of gold particles globally or in-cell compartments was counted in 35 fields (10 μm^2^) per image. For each combination (mock-inoculated plants and TuMV-inoculated Col-0, *Atgstu19*, and *Atgstu24* plants), gold particles from 200 photographs were counted to determine the presence of glutathione or TuMV content.

## 5. Conclusions

Many works have demonstrated that glutathione and glutathione metabolism enzymes play an important role under different plant stress conditions, especially pathogen challenges. However, glutathione metabolism in specific plant–virus pathosystems can be differentially modulated, and our knowledge about this is far from sufficient. Therefore, in this work, the response of *Atgstu19* and *Atgstu24* knockout mutants to TuMV inoculation was examined and compared. Even though the general function of GSTUs has been documented, the potential function of AtGSTU-mediated response in TuMV remains unexplored so far.

In *Atgstu24*–TuMV interactions, a more intense reduction in virus titers and a significant reduction in the *TuMV-CP* relative gene expression level were documented, compared with Col-0–TuMV and *Atgstu19*–TuMV. Ultrastructural analyses confirmed the localization of rare virus particles in vacuoles of inoculated leaf tissues and the lack of virus cytoplasmic inclusions and organelle alterations. Therefore, we postulated a resistance-like reaction to TuMV in *Atgstu24*, followed by a dynamic increase in the reduced GSH and total glutathione contents with high GST and GR activity. Importantly, in *Atgstu24*–TuMV, AtGSTU19 and AtGSTU13 contributed to a resistance-like reaction, whereas AtGSTU1 only participated in the early step of interaction until the symptoms appeared. Furthermore, glutathione activated plant defense, reduced the virus content, and decreased the potential damage to the host plant cell. Additionally, when a resistance-like reaction was induced in *GSTU24* knockout mutants, AtGSTU24 may suppress plant resistance.

On the contrary, in *Atgstu19*–TuMV interactions, induction of virus infection typical for the susceptible reaction was observed. Moreover, AtGSTU1 and AtGSTU24 highly correlated with susceptibility, but AtGSTU13 with symptom development at the 7 dpi time point, similar to Col-0 wild-type. Furthermore, the GSH content was only upregulated until 7 dpi and the total glutathione content until 3 dpi. However, the GSSG content decreased earlier and quicker (at 3 dpi), compared with Col-0, accompanied by GST and GR activity downregulation between 7 and 14 dpi. Therefore, taking into account much more intense virus content and glutathione modulation, enhanced susceptibility was observed in the *Atgstu19*–TuMV pathosystem. Additionally, when *GSTU19* knockout mutants revealed susceptible reaction, resistant plants may require AtGSTU19. A comparison of *A. thaliana* knockout mutant–TuMV interaction indicated that different GSTUs can be involved in the differential modulation of plant response to TuMV inoculation. Further molecular and cellular studies on overexpressing GST mutants are needed to elucidate the possible role of other active components in the TuMV–*A. thaliana* pathosystem.

## Data Availability

Not applicable.

## Supplementary Materials

The following supporting information can be downloaded at: https://www.mdpi.com/article/10.3390/ijms231911531/s1.
