# Supplementary material for: AtGSTU19 and AtGSTU24 as Moderators of the Response of Arabidopsis thaliana to Turnip mosaic virus
Source: Int J Mol Sci. 2022 Sep 29;23(19):11531. doi: 10.3390/ijms231911531 (PMC9570173; doi:10.3390/ijms231911531)
Supplement: Supplementary file 1 [file ijms-23-11531-s001.zip › ijms-1939329-supplementary.pdf]

Table S1. TuMV detection assessment using DAS-ELISA on *Arabidopsis thaliana* wild type and mutant leaves at 3 and 7 dpi. Values presented are mean optical density (OD_{405nm}) values. Absence of virus is marked by (-). Presence of TuMV (+) is confirmed in samples with mean OD_{405nm} above estimated cut off point: 0.1412.

**Sample**	**Mean OD_{450nm}**	**Presence (+)/absence of the virus (-)**
buffer	0.0001	-
mock-inoculated Col-0 (3dpi)	*0.0305*	-
mock-inoculated *Atgstu19* (3dpi)	*0.0213*	-
mock-inoculated *Atgstu24* (3dpi)	*0.0200*	-
TuMV-inoculated Col-0 (3dpi)	*0.8820*	+
TuMV-inoculated *Atgstu19* (3dpi)	*0.7519*	+
TuMV-inoculated *Atgstu 24* (3dpi)	*0.5200*	+
mock-inoculated Col-0 (7dpi)	*0.0501*	-
mock-inoculated *Atgstu19* (7dpi)	*0.0521*	-
mock-inoculated *Atgstu 24* (7dpi)	*0.0402*	-
TuMV-inoculated Col-0 (7dpi)	*1.425*	+
TuMV-inoculated *Atgstu19* (7dpi)	*3.321*	+
TuMV-inoculated *Atgstu24* (7dpi)	*0.693*	+
mock-inoculated Col-0 (14dpi)	*0.0600*	-
mock-inoculated *Atgstu19* (14dpi)	*0.0632*	-
mock-inoculated *Atgstu 24* (14dpi)	*0.0606*	-
TuMV-inoculated Col-0 (14dpi)	*1.939*	+
TuMV-inoculated *Atgstu19* (14dpi)	*4.026*	+
TuMV-inoculated *Atgstu24* (14dpi)	*0.299*	+

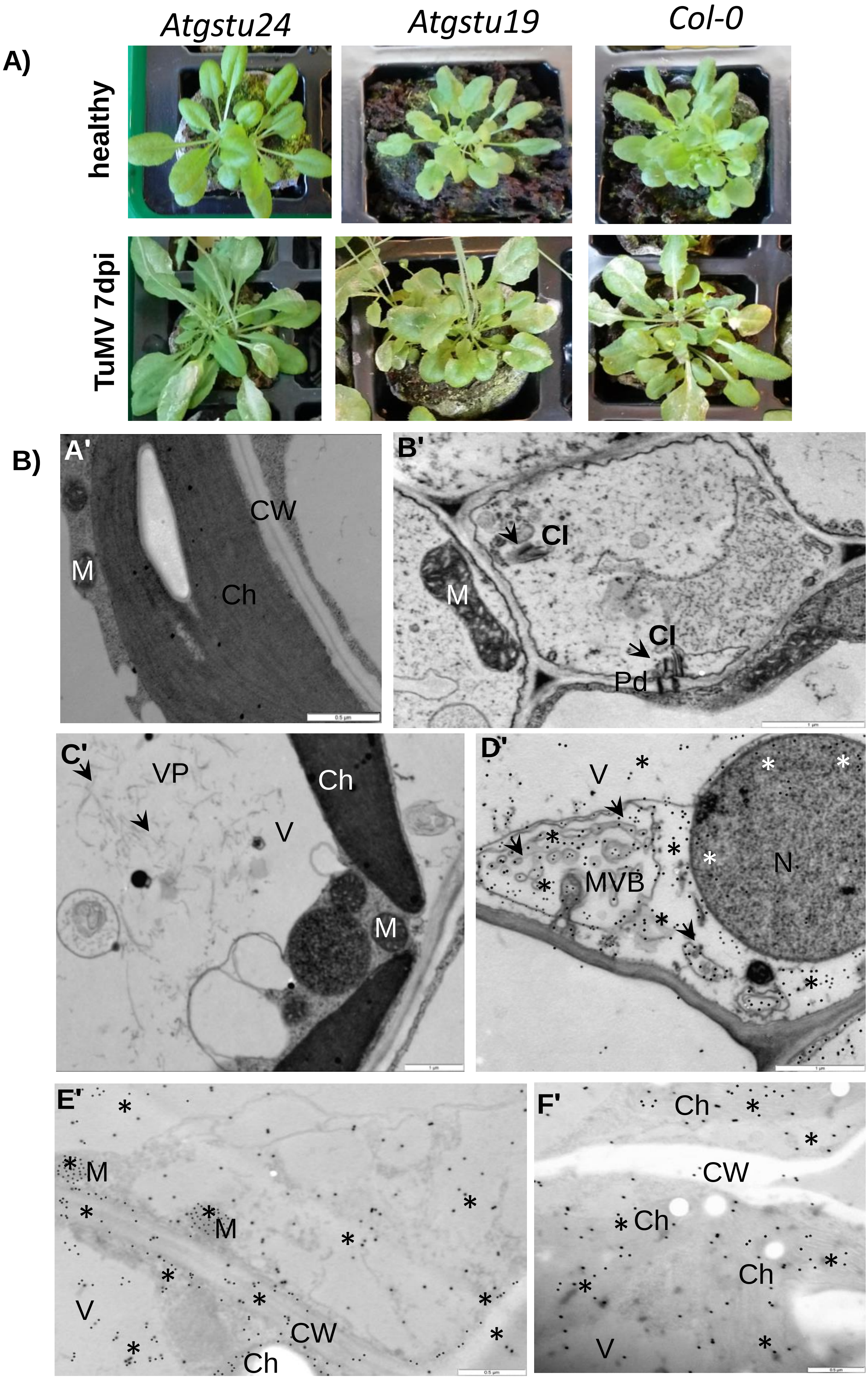

Supplementary Figure S1

Supplementary Figure S1
Panel A) - *Arabidopsis thaliana Atgstu24*, *Atgstu19* knockout mutant plants and Col-0 wild type plants: healthy plants, and 7 dpi afer TuMV inoculation;

Panel B) – Ultrastructural changes in Col-0 wild type inoculated leaves: **(A')** Unchanged mesophyll cel from Col-0 mock-inoculated leaf. CW-cel wall, Ch-chloroplast, M-mitochondria. Scale bar 0.5μm; **(B')** TuMV cytoplasmic inclusions (CI, arrows) in phloem parenchyma cells 7 days after TuMV inoculation. M-mitochondria, Pd-plasmodesmata. Scale bar 1μm. **(C')** Virus particles (VP, arrows) in vacuole (V) of mesophyll cell. Ch-chloroplast, M-mitochondria. Scale bar 1μm. **(D')** Gold particles indicating TuMV (*) in multivesicular bodies (MVB, arrows) and in nucleus (N) in mesophyll cell 7 dpi after TuMV inoculation. Scale bar 1μm. **(E')** Gold granules indicating GSH (*) deposition along cell wall (CW), in mitochondria (M) and chloroplasts (Ch) in phloem Col-0 7dpi TuMV-inoculated leaf. Scale bar 0.5 μm. **(F')** GSH (*) deposition in changed chloroplasts (Ch) in Col-0 mesophyll cell 14 days after TuMV inoculation. Scale bar 0.5 μm.

Table S2. Heatmap of PCC ***for AtGSTU1 normalized expression (based on*** *AtEf1α* and *AtF-Box****) and TuMV levels*** *in* virus-inoculated **Col-0**, ***Atgstu19*** and ***Atgstu24*** **plants from 3 to 14 dpi.** *PCC matrix values are presented pairwise for* specific cell compartments in specific time dpi and marked with *colors, from very dark blue (PCC = 1) to bright blue (PCC = -1).*

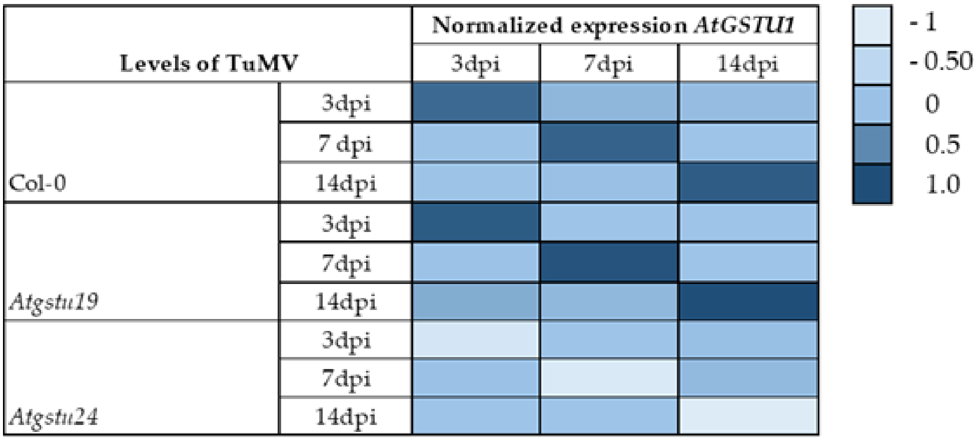

Table S3. Heatmap of PCC ***for AtGSTU13 normalized expression (based on*** *AtEf1α* and *AtF-Box****) and TuMV levels*** *in* virus-inoculated **Col-0**, ***Atgstu19*** and ***Atgstu24*** **plants from 3 to 14 dpi.** *PCC matrix values are presented pairwise for* specific cell compartments in specific time dpi and marked with *colors, from very dark blue (PCC = 1) to bright blue (PCC = -1).*

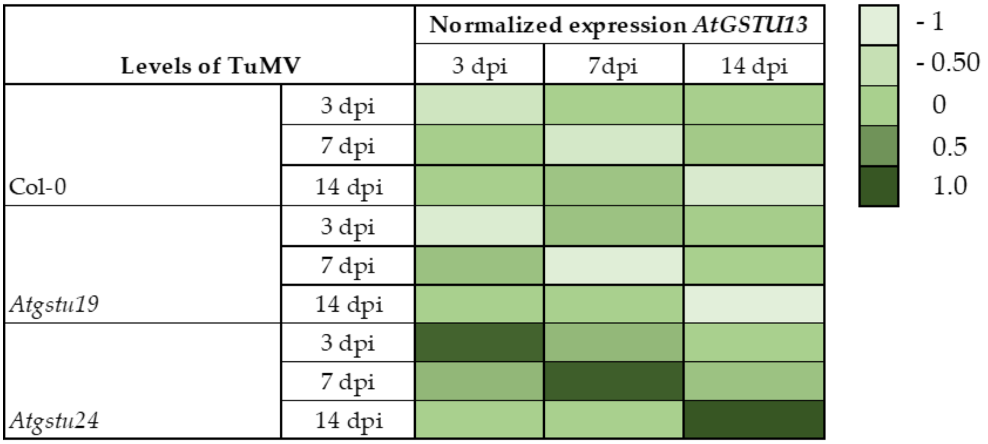

Table S4. Heatmap of PCC ***for AtGSTU19 normalized expression (based on*** *AtEf1α* and *AtF-Box)* ***and TuMV levels*** *in* virus-inoculated **Col-0**, ***Atgstu19*** and ***Atgstu24*** **plants from 3 to 14 dpi.** *PCC matrix values are presented pairwise for* specific cell compartments in specific time dpi and marked with *colors, from very dark blue (PCC = 1) to bright blue (PCC = -1).*

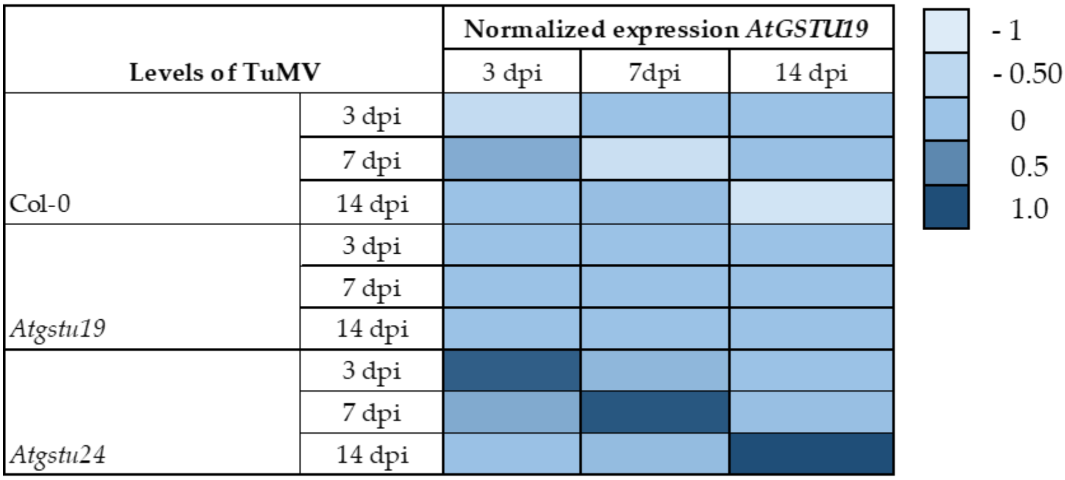

Table S5. Heatmap of PCC ***for AtGSTU24 normalized expression (based on*** *AtEf1α* and *AtF-Box)* ***and TuMV levels*** *in* virus-inoculated **Col-0**, ***Atgstu19*** and ***Atgstu24*** **plants from 3 to 14 dpi.** *PCC matrix values are presented pairwise for* specific cell compartments in specific time dpi and marked with *colors, from very dark blue (PCC = 1) to bright blue (PCC = -1).*

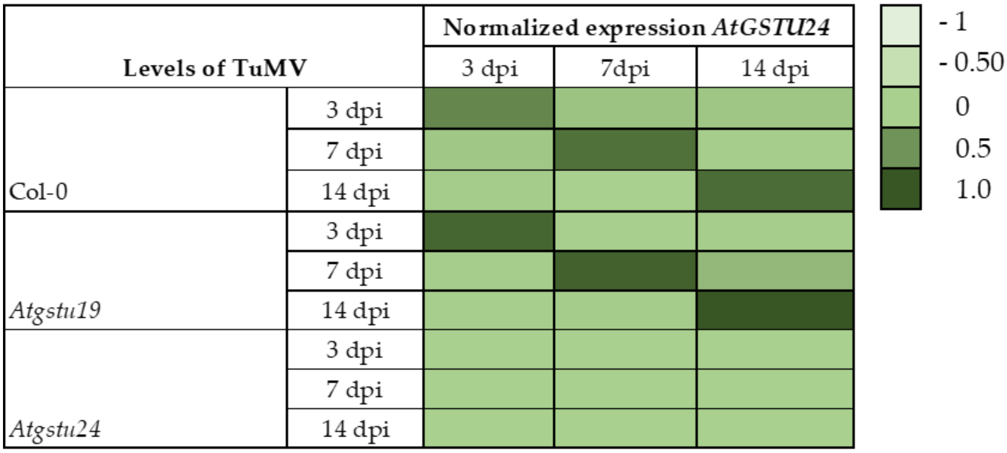

Table S6. Primer sequences for RT-qPCR analyses

Genes	Forward Primer	Reverse Primer	Concentration in Reaction (µM)
		Invastigated	
TuMV-CP	5′-CCGGAATTCATGRTITGGTGYATIGAIAAYGG-3′	5′-CGCGGATCCGCIGYYTTCATYTGIRIIWKIGC-3′	0.5
AtGSTU1	5′-GCAGTGAGGGGATGTATTC-3′	5′-TTTCGTAGGCAAGAAGTATCT-3′	0.5
AtGSTU13	5′-CGCAAAGCAAAAGTTCAATGT-3′	5′-TGGCACAAAACACAGACAAAT-3′	0.5
AtGSTU19	5′-ATGATGCTCAGAGGAAGGTG-3′	5′-ATAGCCAAAGTCATCGCCAC-3′	0,5
AtGSTU24	5′-AAGGTGAGGAGCAAGAAGCA-3′	5′-ACATACCCAAAAGTTTCGTCTC-3′	0.5
		Reference	
AtEF1a	5′-CACCACTGGAGGTTTTGAGG -3′	3′-TGGAGTATTTGGGGGTGGT -5′	0.5
AtF-Box	5′-GCTTGCACACGCCATATCAAT-3′	3′-TGGATTTTACCACCTTCCGCA-5′	0.5

Table S7. Conditions of the RT-qPCR for the reference genes (*).

Program	Parameters
Preliminary denaturation	95 °C for 5 min
Amplification (35 cycles)	95 °C for 10 s<br>58 °C for 10 s<br>72 °C for 20 s *
Melting curve	65–95 °C; 0.1 °C/s
